# An exploratory investigation of the CSF metabolic profile of HIV in a South African paediatric cohort using GCxGC-TOF/MS

**DOI:** 10.1007/s11306-024-02098-y

**Published:** 2024-03-01

**Authors:** Anicia Thirion, Du Toit Loots, Monray E. Williams, Regan Solomons, Shayne Mason

**Affiliations:** 1 Department of Biochemistry, Human Metabolomics, Faculty of Natural and Agricultural Sciences, North-West University, Potchefstroom, North West South Africa; 2 Department of Paediatrics and Child Health, Faculty of Medicine and Health Sciences, Stellenbosch University, Tygerberg, 7505 South Africa

**Keywords:** Paediatric, HIV, Cerebrospinal fluid (CSF), HIV-associated neurocognitive disorders (HAND), GCxGC-TOF MS

## Abstract

**Introduction:**

Because cerebrospinal fluid (CSF) samples are difficult to obtain for paediatric HIV, few studies have attempted to profile neurometabolic dysregulation.

**Aim and objective:**

The aim of this exploratory study was to profile the neurometabolic state of CSF from a South African paediatric cohort using GCxGC-TOF/MS. The study included 54 paediatric cases (< 12 years), 42 HIV-negative controls and 12 HIV-positive individuals.

**Results:**

The results revealed distinct metabolic alterations in the HIV-infected cohort. In the PLS-DA model, 18 metabolites significantly discriminated between HIV-infected and control groups. In addition, fold-change analysis, Mann–Whitney U tests, and effect size measurements verified these findings. Notably, lactose, myo-inositol, and glycerol, although not significant by p-value alone, demonstrated practical significance based on the effect size.

**Conclusions:**

This study provided valuable insights on the impact of HIV on metabolic pathways, including damage to the gut and blood–brain barrier, disruption of bioenergetics processes, gliosis, and a potential marker for antiretroviral therapy. Nevertheless, the study recognized certain constraints, notably a limited sample size and the absence of a validation cohort. Despite these limitations, the rarity of the study’s focus on paediatric HIV research underscores the significance and unique contributions of its findings.

**Supplementary Information:**

The online version contains supplementary material available at 10.1007/s11306-024-02098-y.

## Introduction

According to the 2022 UNAIDS report, around 1.7 million children under 15 years of age were living with HIV in 2021. Antiretroviral therapy (ART) has significantly delayed the progression of HIV disease, so that infected individuals lead near normal lives (Kaur et al., 2020). However, even with virologic suppression, HIV infection is associated with chronic immune activation, metabolic aberrations, and higher rates of comorbidities such as cardiovascular problems, kidney failure, diabetes, cancer, and HIV-associated neurocognitive disorders (HAND) (Cassol et al., 2014; Kaur et al., 2020; Ziegler et al., 2017). HIV infiltrates the central nervous system (CNS) compartment via infected monocytes and CD4 + T lymphocytes and subsequently infects the perivascular macrophages and microglia (Cochrane et al., 2022; Gonzalez-Scarano & Martin-Garcia, 2005). Once within the CNS, HIV can trigger HAND by dysregulating normal neuroimmunity and inducing chronic inflammation that subsequently disrupts normal neurometabolism.

Although the incidence of debilitating HIV-associated dementia (HAD) has decreased with the introduction of ART, HAND remains a persistent problem, even with viral suppression (Gonzalez-Scarano & Martin-Garcia, 2005; Kaul & Lipton, 2006). Data informing our understanding of HAD and HAND are predominantly from adult cohorts. However, children appear to be more vulnerable to HIV’s impact on the CNS, since untreated children show a higher prevalence of CNS dysfunction compared to untreated adults (Gonzalez-Scarano & Martin-Garcia, 2005). In paediatric HIV + populations, neurodevelopmental delays and cognitive impairments are common, encompassing various areas such as vision, language, attention, memory, learning, speech, hearing, and fine motor functioning (Vreeman et al., 2015; Yadav et al., 2017). There are limited paediatric-specific data regarding the mechanisms of HIV pathogenesis in the brain, especially in terms of metabolomics, due to the challenge of acquiring cerebrospinal fluid (CSF) for such investigations. This exploratory study aimed to create an untargeted metabolomics dataset using GCxGC-TOF/MS to investigate the metabolic basis of HIV-induced neuropathogenesis in the CSF of South African paediatric participants.

## Materials and methods

### Sample collection

This retrospective study was conducted at Tygerberg Hospital, Cape Town, South Africa. CSF samples were collected via a lumbar puncture from 54 paediatric cases (< 12 years of age), of which 42 were HIV negative (controls) and 12 were HIV positive (HIV +). All CSF samples were retrospectively collected for routine diagnostic purposes between 2010 and 2017 from children suspected of meningitis. All children underwent a comprehensive clinical assessment. CSF evaluation included macroscopic appearance, total and differential cell count, protein, glucose, chloride, Gram stain, India ink examination, auramine’O ‘ fluorescence microscopy, culture for pyogenic bacteria, culture for *Mycobacterium tuberculosis (M.tb)*, GenoType MTBDR*plus* assay, and GeneXpert MTB/RIF**®** assay. The screening included HIV enzyme-linked immunosorbent assay (ELISA) in children with or without maternal HIV exposure or where the HIV ELISA screening test was positive, HIV DNA polymerase chain reaction (PCR) was performed. Written, informed consent and/or assent were obtained for CSF samples from all participants or their legal guardians for research purposes. The study was approved by the Health Research Ethics Committee (HREC) of Stellenbosch University, Tygerberg Hospital (ethics approval no. N16/11/142), the Western Cape Provincial Government, as well as by the HREC of the North-West University (NWU), Potchefstroom campus (ethics approval no. NWU-00,063-18-A1). All the CSF samples were kept frozen and transported overnight to the Centre for Human Metabolomics at the Potchefstroom campus of the North-West University, where they were stored in a dedicated −80 °C freezer in a Biosafety Level 3 (BSL3) laboratory until analysis. Samples were filtered using Amicon Ultra-2 mL 10,000 MWCO centrifugal filters at 4500×*g* for 20 min to sediment all macromolecular components and yield a sterile, non-infectious sample for further analysis. Of the filtrate, 50 µl was aliquoted for GCxGC-TOF/MS analysis and 20 µl was aliquoted for a pooled QC sample.

### Participant demographics

The prevalence of HIV co-infection among paediatric meningitis suspects in the hospital is relatively low (Solomons et al., 2016). Despite limited sample availability, this provides a rare opportunity to investigate the influence of HIV on the CNS compartment in children. Four of the HIV + participants were exposed to ART. All of them were on a combination of nucleoside reverse transcriptase inhibitors (NRTIs), including Stavudine, Lamivudine, and Abacavir, as well as protease inhibitors such as Lipinovir/Ritonavir. For obvious ethical reasons, the control group was not entirely healthy and included participants with other neurological complications. Hence, the variables found to discriminate between the HIV + and the control group excluded metabolites that arise in a generic response to neurological injury or inflammation. Thus, the variables selected were specific to HIV infection. Table 1 summarizes the collected demographics and clinical information collected from this study cohort. It is important to note that all participants in this study were confirmed negative for meningitis by normal cerebrospinal fluid findings but a clinical symptom such as meningeal irritation, clinically manifested by neck stiffness, was confirmed in one HIV + participant, which was not excluded from this study. This has been documented, sometimes together with headache, fever, nausea and vomiting as early manifestations of HIV infection. (Siddiqi et al., 2015). Pneumonia and upper respiratory-tract infections are other potential causes of neck stiffness in children with HIV-infection (Oostenbrink et al., 2001). The virus damages the integrity of the BBB by various mechanisms, and it is therefore important to note any metabolic changes that might be associated with this clinical presentation.

**Table 1:** Clinical metadata

	HIV + (N = 12)	Control (N = 42)	HIV vs Controls (p-value)
Male/female, n (%)	8/4 (66.7/33.3)	28/14 (66.7/33.3)	1
Age in months			
Average [Q1-Q3]	21 [7–30]	34 [14–44]	0.27
Clinical symptoms, n (%)			
Fever	9 (75)	24 (57.1)	0.49
Cough	7 (58.3)	11 (26.2)	0.06
Vomiting	4 (33.3)	22 (52.4)	0.59
Diarrhoea	5 (41.7)	7 (16.7)	0.11
Weight loss	1 (8.3)	4 (9.8)	1
Headache	2 (16.7)	9 (21.4)	1
Decreased consciousness	1 (8.3)	10 (23.8)	0.49
Concurrent infections, n (%)			
Pneumonia U	4 (33.3)	8 (19)	0.54
Urinary tract infection	1 (8.3)	0	0.51
Gastroenteritis	2 (16.7)	2 (4.8)	0.46
Varicella zoster infection	0	1 (2.4)	1
Flue	0	1 (2.4)	1
Tonsillitis	0	3 (7.1)	0.79
Abscesses	0	1 (2.4)	1
*M.tb* (non-CNS) infection	4 (33.3)	2 (4.8)	0.02
Neurological symptoms, n (%)			
Seizures	3 (25)	17 (41.5)	0.76
Raised intracranial pressure	1 (8.3)	0	0.48
Meningeal irritation	1 (8.3)	4 (9.8)	1
Brainstem dysfunction	0	1 (2.4)	1
Infarction	2 (16.7)	1 (2.4)	0.17
Basal enhancement	0	3 (7.3)	0.90
Hydrocephalus	1 (8.3)	1 (2.4)	0.85
Hemiplegia	2 (16.7)	1 (2.4)	0.12
Abnormal MRI	0	0	
CSF cell count (cells per µL CSF)			
Erythrocytes (median [IQR])	0 [0–121]	0 [0–4.3]	0.26
Leukocytes (median [IQR])	1 [1–2]	0 [0–1]	0.13
PMNs (median [IQR])	0	0	
Lymphocytes(median [IQR])	1 [1–2]	0 [0–1]	0.10
CSF protein (g/L) (median [IQR])	0.2 [0.2–0.3]	0.19 [0.14–0.27]	0.51
CSF glucose (mmol/L) (median [IQR])	3.4 [3.1–4.5]	3.9 [3.5–4.3]	0.37
CD4 count (cells/uL) (median [IQR])	302 [10.8–593]	N/A	
CD4 count (%) (median [IQR])	21.5 [16.5–29.5]	N/A	
Viral load Log10(copies/mL)(median [IQR])	5.87 [5.30–7]	N/A	
On cART, n (%)	4 (33.3)	0	
Details of cART	Kaletra, Stavudine, 3TC- duration 4 months Kaletra, Abacavir, Ritonivir, 3TC- duration 25 days		
	Kaletra, Abacavir, Ritonivir, 3TC- duration 25 days		
	Abacavir, Kaletra, 3TC- duration 10 months		
	Lamivudine, Abacavir, Lipinovir/Ritonavir duration 21 days		

### Chemicals

3-Phenylbutyric acid, methoxyamine hydrochloride, pyridine and N,O-bis(trimethylsilyl)-trifluoroacetamide (BSTFA) with 1% trimethylsilyl chloride (TMCS) were purchased from Sigma-Aldrich (St. Louis, Missouri, USA). Methanol and chloroform were purchased from Honeywell International Inc. (Muskegon, Michigan, USA).

### Sample preparation

Before sample preparation, an internal standard stock solution (50ppm) of 3-phenylbutyric acid dissolved in methanol and 15 mg/mL methoxyamine hydrochloride in pyridine was prepared. The sample preparation method entailed adding 50 µl of internal standard solution to 50 µL of each CSF sample and drying at 40 °C under a light stream of nitrogen for 45 min. Thereafter, 50 µL of methoxamine hydrochloride was added to each dried CSF sample and vortexed for 30 s. Hereafter, incubation of the samples at 50 °C for 90 min. Finally, 80 µL of BSTFA with 1% TMCS was added and incubated at 60 °C for 60 min. Each sample was transferred to a GC–MS vile containing a vile insert and capped.

### GCxGC‑TOF/MS analysis and processing

The samples were randomized across each batch with QCs placed intermittently in an auto-sampler. All samples were analysed on a Pegasus 4D GCxGC-TOF/MS system (LECO Africa (Pty) Ltd, Johannesburg, South Africa) fitted with an Agilent 7890A GC. One microlitre of each sample was injected with a 1:5 split ratio, with the injector temperature fixed at 270 °C for the duration of the analysis. Purified helium was used as a carrier gas and set to a constant flow rate of 1 mL/min. Compound separation was achieved with a Restek Rxi-5Sil MS primary capillary column (30 m, 0.25 µm film thickness, and 250 µm internal diameter). An initial temperature was set at 50 °C, followed by a 4 °C/min temperature increase until a final temperature of 300 °C was reached and maintained for 2 min. The Rxi-17 secondary capillary column (0.9 m, 0.1 µm film thickness and 100 µm internal diameter) was set to an initial temperature of 85 °C, followed by a 4.5 °C/min temperature increase until a final temperature of 300 °C and maintained for 4.5 min. The thermal modulator pulsed cold and hot streams of nitrogen gas every 3 s for 0.5 s. No mass spectra were recorded for the first 530 s to exclude solvent detection. The transfer line was set to a temperature of 270 °C, and the ion source was fixed at 250 °C. The detector voltage was 150 V, and the filament bias was −70 eV. Mass spectra were acquired at a rate of 200 spectra in a range of 50–800 m/z.

Data processing was performed using LECO Corporation’s ChromaTOF software (version 4.32). Mass spectral deconvolution was performed at a signal-to-noise ratio of 20, with a minimum of five apex peaks. Retention time shifts were corrected across all samples by aligning identical mass spectra that displayed similar retention times. Peak identification was made possible by comparing the characteristic mass fragmentation patterns and retention times with a library of previously injected standards.

### Statistical analyses

We used the Chi-squared test to evaluate group differences in clinical presentation. A total of 216 compounds were detected during analysis. Data cleanup involved normalizing compounds to 3-phenylbutyric acid and calculating their relative concentrations. Variables with no group variation and more than 50% zero values in both groups were removed. Batch correction was performed with the QC samples using quantile equating. Statistical analyses were performed in MetaboAnalyst 5.0 and IBM SPSS Statistics (v. 28.0.1.1). Missing values were replaced with 1/5 of the minimum positive value for each variable. Based on the median absolute deviation, variables with QC relative standard deviation values greater than 40% were removed. Data were normalized with square root transformation and autoscaling. Univariate analyses included fold change (FC) and Mann Whitney U tests with FC < 0.5 or > 2.0 and p-value < 0.1 considered significant. Effect sizes (d > 0.5 practically significant) calculated in SPSS. Multivariate methods included principal component analysis (PCA) and partial least squares–discriminant analysis (PLS-DA), with variables important in projection (VIP) values > 1.0 considered significant. Generalized linear modelling was employed to assess the influence of age on the statistically significant metabolites. Lastly, for the HIV + group, Spearman’s rho correlations were performed in SPSS for the statistically significant metabolites and the continuous clinical features, while point-biserial correlations were performed for metabolites and dichotomous clinical features. The correlation data are included as supplementary material (Tables S1 and S2).

## Results

**Fig. 1:**
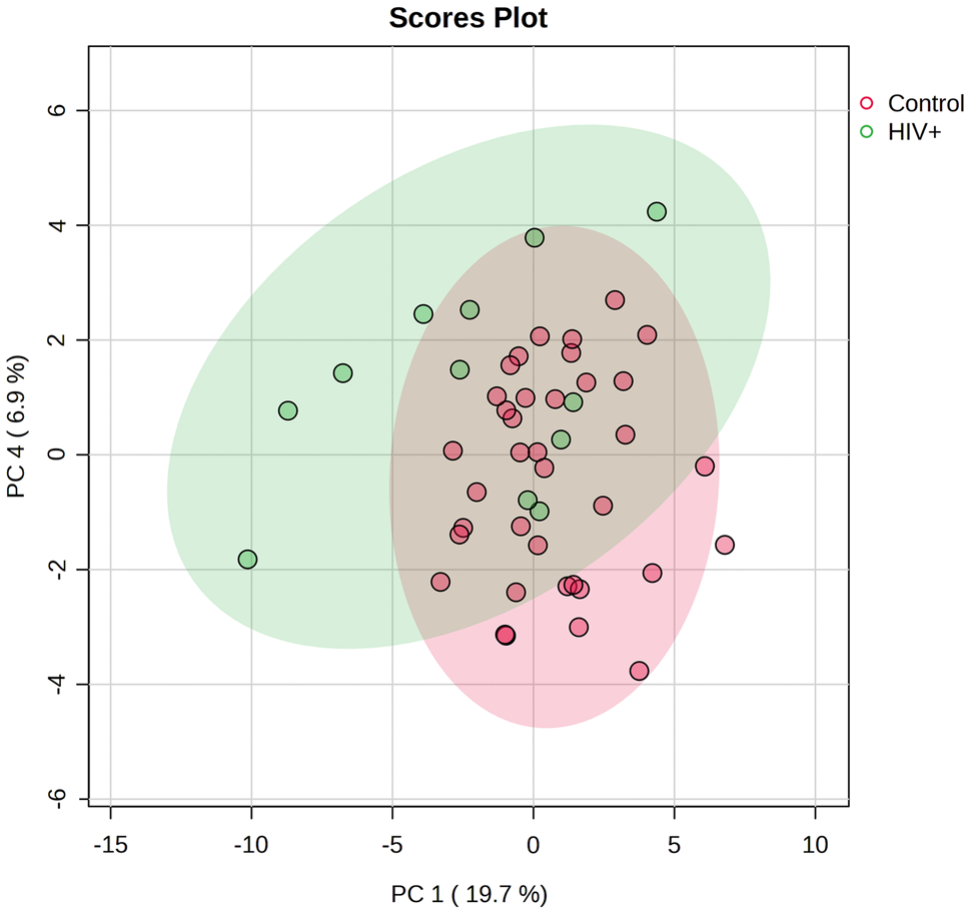
PCA scores plot of PC1 and PC4, indicating a degree of natural separation between the HIV + (green) and control (red) groups, as well as a greater degree of inter-individual variation within the HIV + group

No statistically significant differences were observed in clinical presentation between the two groups. Five control samples appeared outside the PCA 95% confidence interval and were removed from the analysis. Visual inspection of the PCA scores plot (Fig. 1) indicates an overlap between the HIV + and control groups. However, there is some degree of natural separation between the HIV + and control groups at PC1 vs PC4. Furthermore, more inter-individual variation occurs within the HIV + group.

**Fig. 2:**
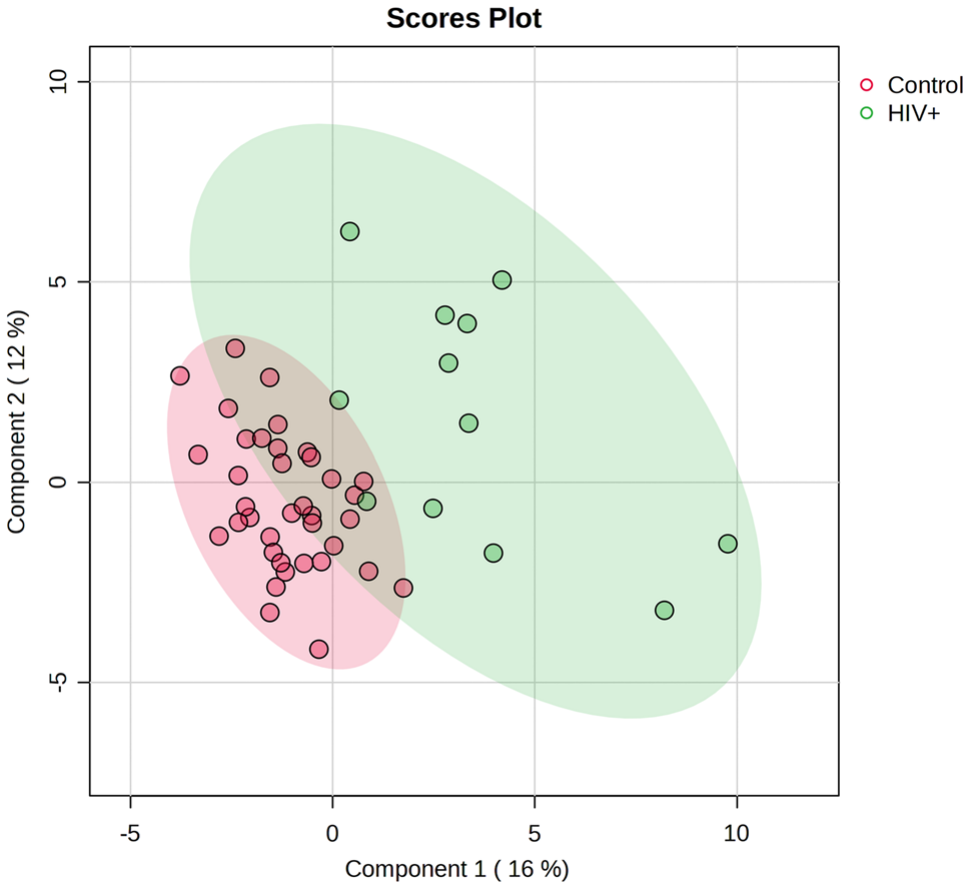
PLS-DA scores plot indicating group differentiation between the HIV + (green) and control (red) groups. This model was cross-validated and had an accuracy measure of 0.857, an R2-value of 0.855, and a Q2-value of 0.468. A permutation test based on prediction accuracy revealed a p-value of 0.026

The PLS-DA model (Fig. 2) differentiated between the HIV-infected and control groups and cross-validated with an accuracy measure of 0.857, an R2-value of 0.855, and a Q2-value of 0.468. A permutation test on the PLS-DA model showed prediction accuracy and the data were not overfitted, with a p-value of 0.026. Seventeen metabolites significantly differentiated the HIV + and control groups based on PLS-DA VIP values > 1.0.

Seven metabolites had a fold-change (FC) less than 0.5 or greater than 2 in the HIV + group comparatively. Sixteen metabolites differed significantly between the HIV + and control groups based upon the Mann–Whitney U test, corrected for multiple testing, with a threshold p < 0.1. Eighteen metabolites differed significantly between the HIV + and control groups based upon an effect size with a threshold d > 0.5. Metabolites with a p-value < 0.1 and d-value > 0.5 are the cut-off criteria here for the metabolic characterization of HIV, with PLS-DA VIP > 1.0 used to confirm these results. Three metabolites did not have significant p-values (p > 0.1) but did have statistically significant effect sizes (d > 0.5) when comparing the groups; namely, lactose (d > 0.8), myo-inositol (d > 0.8) and glycerol (d > 0.6). For a cohort of this size, the p-value alone is insufficient to determine the significance of an effect. The effect size, on the other hand, provides a measure of the magnitude of an effect and is independent of the sample size (Ialongo, 2016; Sullivan & Feinn, 2012). The effect of chance overshadows the statistical significance for these three metabolites (Ialongo, 2016), but from the effect size it is clear that they do have some practical significance. Generalized linear modelling revealed no statistically significant influence of age on these results. Table 2 summarizes these statistical results. Box plots are included as supplementary data.

**Table 2:** Summary of statistical findings

Metabolites	HIV			Controls			HIV vs Control			
	Average relative conc. (µmol/L)	±	Standard deviation	Average relative conc. (µmol/L)	±	Standard deviation	Fold change (< 0.5 or > 2.0)	Mann–Whitney U (< 0.1)	VIP score (> 1.0)	d-value (> 0.5)
3-Hydroxybutyric acid	0.0275	±	0.0313	0.0881	±	0.1479	0.31	0.0783		0.556
3-Hydroxyisovaleric acid	0.0017	±	0.0019	0.0007	±	0.0004	2.57	0.0078	1.48	0.864
4-Deoxythreonic acid	0.0018	±	0.0013	0.0006	±	0.0002	3.91	0.0031	1.98	1.039
D-Ribose	0.0062	±	0.0030	0.0040	±	0.0012		0.0116	1.51	2.288
Erythritol	0.0373	±	0.0272	0.0193	±	0.0056		0.0031	1.75	1.484
Glyceric acid	0.0201	±	0.0093	0.0132	±	0.0048		0.0302	1.40	2.199
Glycerol	0.4890	±	0.7098	0.1891	±	0.3843	2.59		1.10	0.532
Lactose	0.0019	±	0.0019	0.0006	±	0.0005	12.15		1.67	0.870
Methylphosphonic acid	0.0020	±	0.0008	0.0014	±	0.0006		0.0242	1.18	2.390
Myo-Inositol	0.4868	±	0.4873	0.2632	±	0.1057			1.11	1.183
Pyroglutamic acid	0.0642	±	0.0224	0.0484	±	0.0187		0.0377	1.24	2.538
Ribitol	0.0799	±	0.0311	0.0475	±	0.0109		0.0022	2.00	2.459
Ribonic acid	0.0141	±	0.0094	0.0083	±	0.0029		0.0193	1.47	1.688
Sorbitol	0.0167	±	0.0097	0.0096	±	0.0044		0.0042	1.53	1.665
Threonic acid	0.0433	±	0.0235	0.0258	±	0.0086		0.0031	1.65	1.944
Trehalose	0.0038	±	0.0036	0.0006	±	0.0006	6.33	0.0031	1.44	0.644
Undecylenic acid	0.0049	±	0.0006	0.0058	±	0.0013		0.0113	1.23	4.642
Urea	0.2480	±	0.2082	0.3759	±	0.2225		0.0853	1.37	1.539

**Fig. 3:**
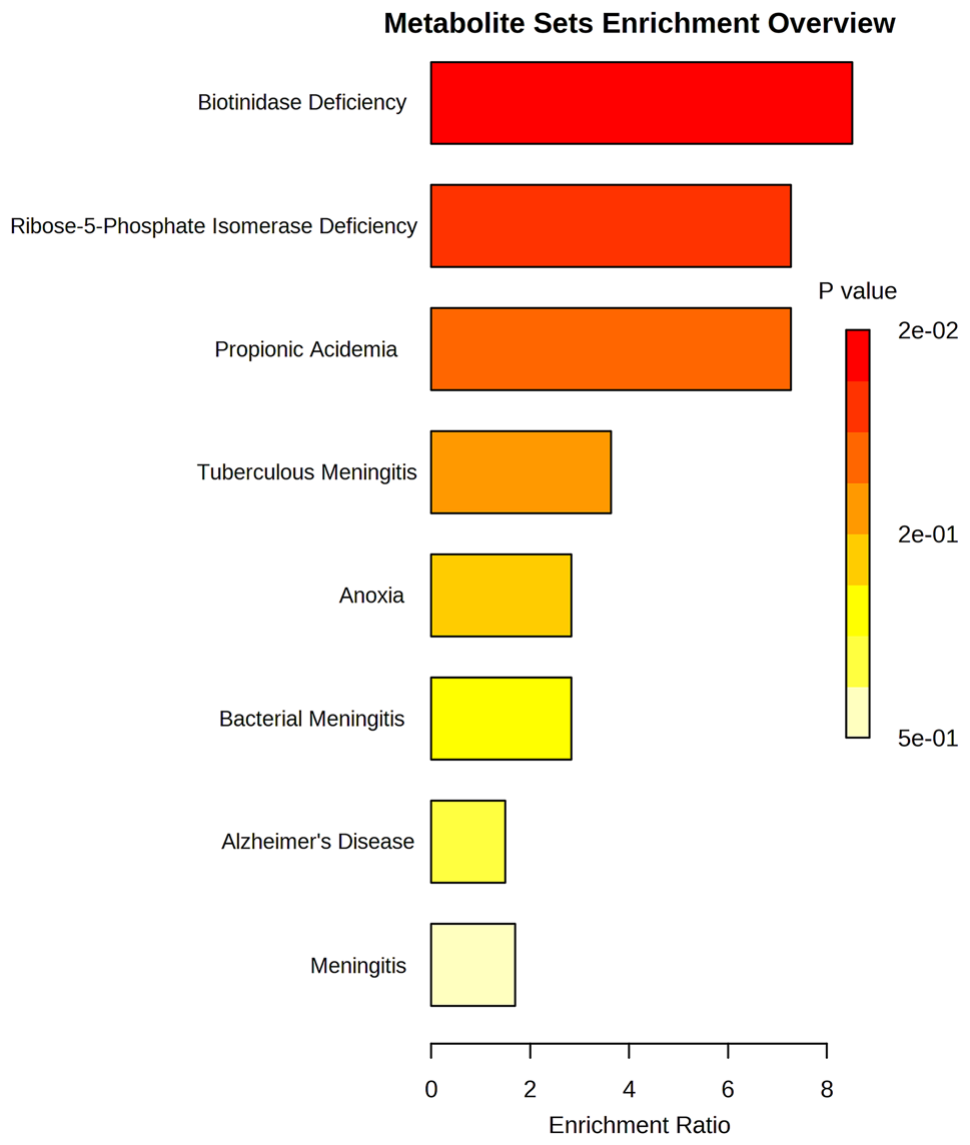
Enrichment analysis of the 18 important CSF metabolites identified for paediatric HIV. This metabolic profile is more closely associated with that of biotinidase deficiency, ribose-5-phosphate isomerase deficiency, and propionic acidemia, and to a lesser degree with various forms of meningitis, anoxia, and Alzheimer’s disease

An enrichment analysis (Fig. 3) of the 18 metabolites that characterise neuroinflammation in CSF collected from this South African paediatric HIV cohort shows that our set of metabolites is closely associated with that of a biotinidase deficiency, ribose-5-phosphate isomerase deficiency, and propionic acidemia. There is also some association with various forms of meningitis, anoxia, and Alzheimer’s disease.

## Discussion

**Fig. 4:**
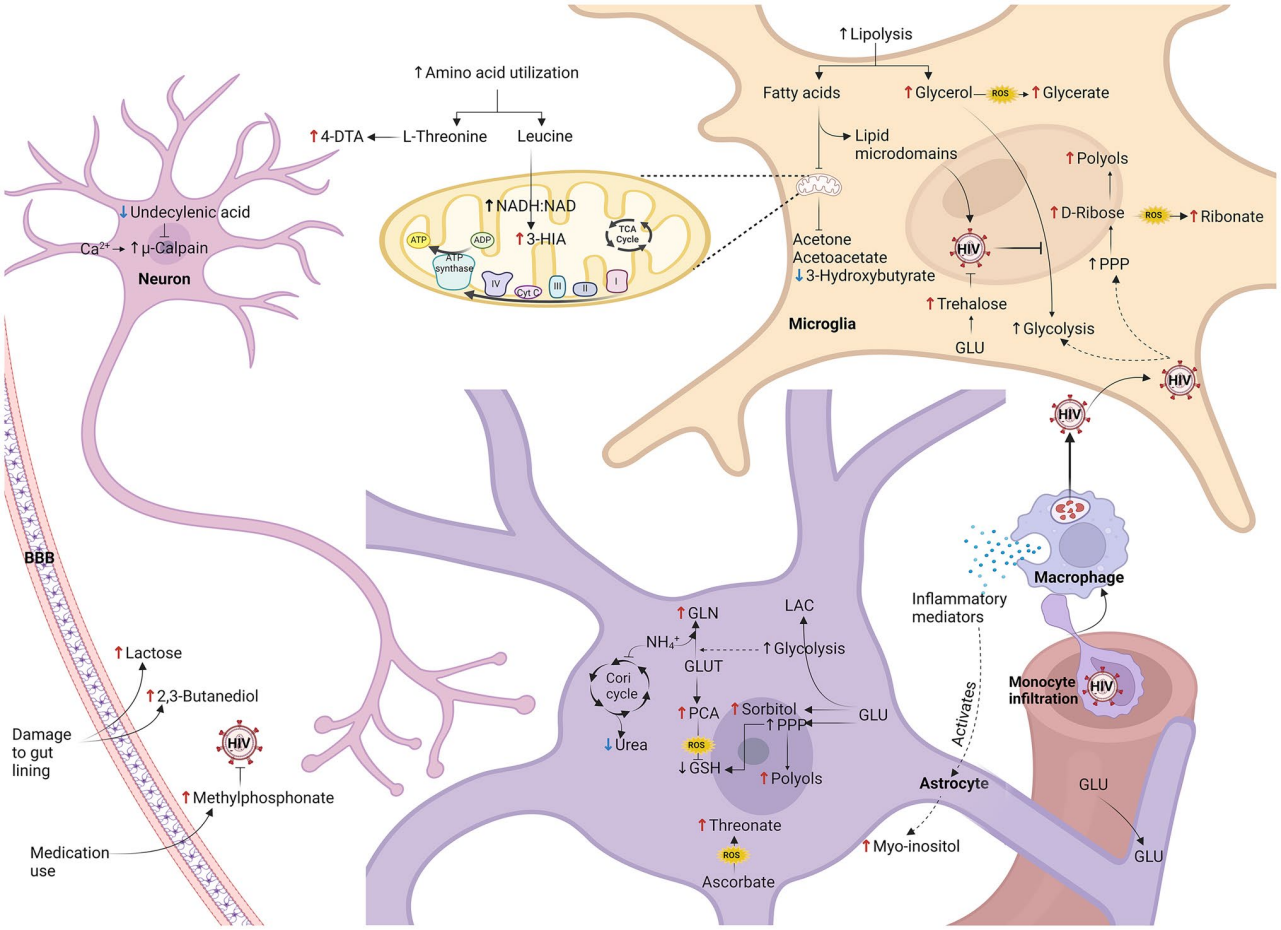
Overview of the proposed neurometabolic pathways affected by HIV infection, with ↑/↓ of significant metabolites indicated. HIV activates cells and induces gliosis. HIV alters metabolic pathways including fatty acid metabolism, the sorbitol pathway, the pentose phosphate pathway, and amino acid metabolism to promote viral replication. Viral replication induces oxidative stress that overwhelms antioxidant systems

Figure 4 provides an overview of all the metabolic pathways discussed below.

### HIV damages the gut lining and blood–brain barrier (BBB)

An early loss of CD4 + T cells from gut-associated lymphoid tissue results in damage to the intestinal lining and subsequent malabsorption of nutrients (Swanstrom & Coffin, 2012). Lactose is metabolized to galactose and glucose by lactase on the brush border of the small intestine, and there was increased lactose in the CSF of this HIV-infected cohort (McLean & Clayton, 1970; Weser et al., 1967). HIV infection has been reported to reduce lactase expression and activity in the small intestinal mucosa (Ullrich et al., 1989). Furthermore, HIV induces chronic cytokine production and increased epithelial permeability and apoptosis (Ziegler et al., 2017), which worsens the leaky gut and disrupts the integrity of the BBB, allowing infiltration of the CNS compartment by HIV-infected immune cells. The disruption of tight junctions leading to epithelial permeability is not entirely corrected with ART (Zicari et al., 2019).

### Dysregulation of bioenergetics pathways

HIV infection is characterised by compromised brain energy metabolism that deteriorates as the disease progresses (Cassol et al., 2014; Deme et al., 2020). After entering the CNS compartment, HIV infects perivascular macrophages and microglia and induces cell activation by reprogramming the bioenergetics pathways, including increasing glycolysis, pentose phosphate pathway (PPP) activity, and fatty acid and amino acid utilisation, producing the metabolic intermediates required for cellular proliferation and viral replication (Cochrane et al., 2022; Shehata et al., 2017). These changes disrupt the microenvironment of the CNS, leading to cellular dysfunction and neurocognitive impairment.

In this paediatric HIV-infected cohort, we observed elevated CSF glycerol levels, indicating increased lipolysis, and reduced 3-hydroxybutyric acid due to fatty acid utilization for viral replication. Higher lipolysis rates provide more glycerol for energy production; however, viral protein R (Vpr) inhibits glycerol’s entry into glycolysis by downregulating triosephosphate isomerase (TPI) and glyceraldehyde-3-phosphate dehydrogenase (G3PD), causing its accumulation and oxidation to glyceric acid (Barrero et al., 2013). The acetyl-CoA released can be catabolized to produce ketone bodies for energy (Kassel et al., 1986). Reduced 3-hydroxybutyric acid in HIV-infected participants was also reported by Keram et al. (2021) and Deme et al. (2022) but contradicts the findings of Cassol et al. (2014). However, ART exposure normalizes serum ketone body levels (Deme et al., 2022). Acetyl-CoA is directed toward fatty acid synthesis, for the generation of longer-chain fatty acids used for viral budding, and post-translational modification of viral proteins via an HIV-induced increase in fatty acid synthase activity (Deme et al., 2022; Kulkarni et al., 2017). HIV-induced dyslipidaemia is associated with cognitive impairment (Keram et al., 2021), possibly partially mediated by reduced bodies of ketones of the CSF, especially in children, because the brain is highly dependent on them during the developmental stages. Tissue culture experiments found that the treatment of neurons with ketone bodies was protective against transactivator of transcription (tat)-induced damage by reducing reactive oxygen species (ROS), lowering intracellular calcium levels, and restoring the mitochondrial membrane potential (Deme et al., 2020).

In this paediatric HIV-infected cohort, we also observed increased pyroglutamic acid levels in the CSF, indicating exhaustion of the antioxidant systems and, consequently, increased production of oxidative metabolites such as threonic acid. Excessive ROS production is a hallmark of HIV infection, even with ART (Banki et al., 1998). Reduced glutathione (GSH) is essential to counteract oxidative damage (Wamelink et al., 2008) and is decreased in HIV-infected individuals (Palmer et al., 2016). HIV reduces the expression of several GSH metabolism enzymes in infected brains and macrophages (Morris et al., 2012; Saing et al., 2016). GSH synthase deficiency leads to γ-glutamylcysteine accumulation, which is then converted to pyroglutamic acid (Lu, 2013). GSH depletion exacerbates intracellular oxidative stress and increases CSF threonic acid levels, an oxidative metabolite of ascorbic acid (vitamin C) degradation (Trezzi et al., 2017).

We also identified elevated CSF levels of D-ribose and four polyols (sorbitol, ribitol, erythritol and myo-inositol), indicating increased flux through the PPP and higher aldose reductase (AR) activity (Van der Knaap et al., 1999). The bottleneck in the glycolytic pathway, created by inhibition of TPI and G3PD, has been shown to commit more glucose to the PPP but can simultaneously increase the sorbitol pathway (Barrero et al., 2013). HIV is believed to increase PPP activity for the sustained production of its own nucleic acids (Deme et al., 2022; Shytaj et al., 2021). Deme et al. (2022) observed higher serum levels of ribose-5-phosphate and erythrose-4-phosphate in HIV-infected individuals, only partially corrected by ART, proving that HIV-induced PPP upregulation is not normalized by ART. HIV accomplishes this by upregulating the expression of glucose-6-phosphate dehydrogenase (G6PD), the first enzyme of the PPP, and reducing transketolase (TKT) expression (Barrero et al., 2013), leading to the accumulation of D-ribose which is progressively metabolized to ribitol by AR and oxidized to ribonic acid (Wamelink et al., 2008). Moreover, malabsorption due to intestinal lining damage and downregulation of microbial genes involved in thiamine biosynthesis leads to a thiamine deficiency in people with HIV (Müri et al., 1999; Park et al., 2021). Reduced expression and activity of TKT, a thiamine dependent enzyme, depletes NADPH, disrupts programmed cell death, and contributes to cancer, neurodegenerative diseases, and AIDS pathologies by affecting T-cell eradication (Banki et al., 1998; Perl, 2007). Therefore, reduced TKT activity or expression potentially contributes to HAND pathogenesis. The sorbitol pathway involves glucose reduction into sorbitol by AR and conversion of sorbitol to fructose by sorbitol dehydrogenase (Stevens et al., 1995). As an organic osmolyte, excessive activation of the sorbitol pathway causes osmotic stress and cell swelling (Lang et al., 1998; Stevens et al., 1995). Osmolar stress further increases AR expression and activity, causing intracellular polyol accumulation (Huang et al., 2013; Perl et al., 2011). Additionally, increased AR activity, which converts NADPH to NADP + and sorbitol dehydrogenase reducing NAD + to NADH, further reduces glutathione levels and creates a redox imbalance (Stevens et al., 1995).

The comparatively higher CSF levels of amino acid catabolites – 3-hydroxyisovaleric acid and 4-deoxythreonic acid in this paediatric HIV-infected cohort indicate changes in leucine and threonine metabolism, respectively. These observations are consistent with other reports of reduced branched-chain amino acids in HIV-infected youth (Ziegler et al., 2017). Elevated 3-hydroxyisovaleric acid, a leucine catabolic product, suggests a possible enzyme deficiency due to an HIV-induced biotin deficiency (Salih, 2018). Impaired 3-methylcrotonyl-CoA carboxylase activity, a biotin-dependent enzyme, results in the accumulation of 3-hydroxyisovaleric acid (Van der Graaf et al., 2010). There is very little information available on 4-deoxythreonic acid, an L-threonine catabolic product; however, increased levels thereof have been reported in diabetic patient biofluids and are likely associated with a disturbance of glucose metabolism (Huang et al., 2013; Kassel et al., 1986). As previously mentioned, HIV increases amino acid utilisation since these substrates are precursors for energy production and biosynthetic processes (Ziegler et al., 2017). Dysregulation of inflammation-related amino acid metabolism contributes to neuronal dysfunction in HIV participants and has been reported to persist despite use of ART (Gostner et al., 2015).

Also detected in this HIV-infected cohort were elevated CSF concentrations of trehalose. Trehalose inhibits viral reproduction in HIV-infected macrophages and CD4 + T cells by inducing macroautophagy and blocks HIV entry by reducing CCR5 expression (Rawat et al., 2020). During periods of starvation, macroautophagy provides nutrient-deprived cells with amino acids and fatty acids (Mehrpour et al., 2010; Ouyang et al., 2009). HIV initially induces macroautophagy to promote replication, but later downregulates it to enhance cell survival and prevent apoptosis of infected cells (Rawat et al., 2020). Excessive self-degradation contributes to HIV pathogenesis and neurodegenerative disorders (Mehrpour et al., 2010; Rawat et al., 2020). Further research on the impact on HIV pathogenesis is required.

Reduced CSF urea levels indicate lower activity of the Cori cycle. Increased proteolysis and a negative nitrogen balance (increased levels of ammonia ions) are metabolic responses to acute infection and are recognized as major contributing factors to HIV-induced wasting (Drӧge et al., 1998; Macallan et al., 1995). Ammonia ions can be utilized for glutamine synthesis or detoxified by the Cori cycle, with subsequent urea production (Drӧge et al., 1998). Another study by our group using ^1^H-NMR found increased glutamine levels in the CSF of this paediatric HIV cohort (Thirion et al., 2024). Therefore, the ammonia ions are likely utilized for glutamine synthesis and lowering urea production.

### HIV induces gliosis

Elevated CSF myo-inositol levels in the paediatric HIV + participants in this study are indicative of glial activation and neuroinflammation (Cassol et al., 2014; Dickens et al., 2015; Kaur et al., 2020) and have been associated with the cognitive regression in HIV + participants, even so with viral suppression. Glial activation results in cell hypertrophy and proliferation, and subsequent movement of myo-inositol across cell membranes regulates changes in the resulting cell volume (Fisher et al., 2002). Furthermore, aberrations of myo-inositol metabolism have previously been confirmed in HIV-infected children, especially in the left frontal brain region (Kaur et al., 2020). Depletion of intracellular myo-inositol reduces the availability of phosphoinositide (PI) signalling, which in turn leads to disruption of intracellular calcium homeostasis, neuronal hyperexcitability, and defects in neurotransmitter release and ion conductance (Lang et al., 1998). Na^+^ and K^+^ ions are essential to maintain membrane potential for neurotransmitter-induced excitement (Croze & Soulage, 2013). Altered PI turnover is associated with impaired Na^+^/K^+^-ATPase activity and impaired nerve conductivity (Croze & Soulage, 2013). Additionally, the damage caused by the disruption of the calcium signalling and homeostasis is aggravated by the reduced levels of undecylenic acid, also seen in this HIV-infected cohort, which confirms this. Undecylenic acid is a neuroprotective monounsaturated fatty acid component of phospholipids from the cell membrane in the brain, which acts by inhibiting μ-calpain, a calcium-activated cysteine protease (Jantas et al., 2015). Undecylenic acid protects neurons against amyloid β (A β), glutamate, and H_2_O_2_-induced cell damage by activating the PI3-K/Akt pathway (Jantas et al., 2015). Reduced levels of undecylenic acid in HIV-infected individuals would allow greater activation of μ-calpain and contribute to the development of their neurocognitive symptoms.

#### ART exposure

The elevated methylphosphonic acid detected in the HIV + cohort may serve as an indicator of ART exposure in this group and needs further investigation. Compounds with phosphonate groups – characterized by a direct P–C bond, are typically used as anti-retroviral phosphate analogues that inhibit enzymes that rely on different phosphate substrates, thereby suppressing viral replication by competitively inhibiting the viral DNA polymerase (Krecmerova et al., 2022).

#### Exposure to ART in four of the HIV + participants make interpreting these results more complicated

Studies indicate that ART influences the metabolic profile in HIV-infected individuals, partially by correcting HIV-induced alterations and by inducing its own metabolic changes. However, in this study, we were unable to identify any subgroups within this HIV + group, including differentiation due to exposure to ART. The current literature suggests that many of the metabolic aberrations observed in this study persist despite ART including the elevated PPP activity, dysregulation of amino acid metabolism, high oxidative stress and alteration of antioxidant systems, and increased myo-inositol levels, indicating persistent gliosis. ART has been shown to correct low ketone body levels, but it does not completely correct altered lipid profiles in HIV-infected participants. In fact, it has been shown to induce hyperlipidaemia in perinatally HIV-infected children (Rhoads et al., 2001). This is the first report of the presence of lactose, sorbitol, 3-hydroxyisovaleric acid, 4-deoxythreonic acid, trehalose, and undecylenic acid in the BBB of people with HIV, so the influence of ART is unknown. However, disruption of the integrity of the intestinal lining integrity is not completely corrected with ART (Zicari et al., 2019), and therefore malabsorption leading to increased lactose, TKT deficiency resulting in increased polyols, and 3-methylcrotonyl-CoA carboxylase deficiency resulting in elevated 3-hydroxyisovaleric acid might not be entirely corrected with ART.

#### Limitations

Considering that this is a paediatric population and the sample type for this study was CSF, which is extremely rare and difficult to obtain, the limitations experienced in the study are not easily overcome. These limitations include: (1) the small sample cohort of HIV + and HIV- paediatric samples, but more so for the HIV + cases (which cannot be easily overcome considering the difficulty in acquiring CSF from paediatric patients, and even more so due to the low occurrence of such HIV cases); (2) the lack of viral load data and wide range of CD4 + /mm^3^ values (although no significant correlations of CD4 + /mm^3^ were observed with any metabolic or other clinical markers); (3) the fact that the control group was not entirely healthy (taking CSF from a healthy paediatric population would be unethical; (4) the lack of a validation cohort (once again not easily mended due to scarcity of prevalence of such cases); (5) insufficient data available to assess the influence of viral load. Nonetheless, very few studies have examined CSF from paediatric HIV cases (Williams et al., 2021), making this study rare and the findings of great value.

## Conclusion

In this exploratory study using an HIV paediatric cohort, we found increased levels of unhydrolyzed lactose in the CSF, supporting previous findings that HIV infection damages the intestinal epithelial barrier and the blood–brain barrier (BBB), increasing its permeability and the movement of metabolites and macromolecules into the CNS compartment. Furthermore, HIV alters neurometabolism in favour of the production of metabolic intermediates required for its replication. Affected metabolic pathways include fatty acids, sorbitol, pentose phosphates, and amino acids. Additionally, viral replication induces oxidative stress in the CNS compartment, overwhelming antioxidant systems and oxidizing accumulating metabolic intermediates and possibly lipid membranes. These metabolic alterations may also be associated with HIV-induced activation of immune responses. Our study substantiates previous reports suggesting that HIV triggers gliosis, a process known to contribute to dysregulation of neuronal function in perinatally infected children, similar to that seen in HIV-infected adults. These findings provide a deeper understanding of the intricate metabolic changes that occur in the CNS during paediatric HIV infection.

### Supplementary Information

Below is the link to the electronic supplementary material.

Supplementary file1 (DOCX 1619 KB)

Supplementary file2 (XLSX 26 KB)

Supplementary file3 (XLSX 32 KB)

Supplementary file4 (DOCX 36 KB)

## Data Availability

The datasets generated for this study can be made available upon request from the supporting author.

## Abbreviations

GLU: Glucose
LAC: Lactate
PPP: Pentose phosphate pathway
GSH: Glutathione
GLUT: Glutamate
GLN: Glutamine
PCA: Pyroglutamate
3-HIA: 3-Hydroxyisovaleric acid
4-DTA: 4-Deoxythreonic acid
BBB: Blood brain barrier

## Declarations

### Resource identification

MetaboAnalyst (RRID:SCR_015539).

IBM SPSS Statistics (RRID:SCR_019096).
