# Supplementary figures and images for: An exploratory investigation of the CSF metabolic profile of HIV in a South African paediatric cohort using GCxGC-TOF/MS
Source: Metabolomics. 2024 Mar 1;20(2):33. doi: 10.1007/s11306-024-02098-y (PMC10907482; doi:10.1007/s11306-024-02098-y)

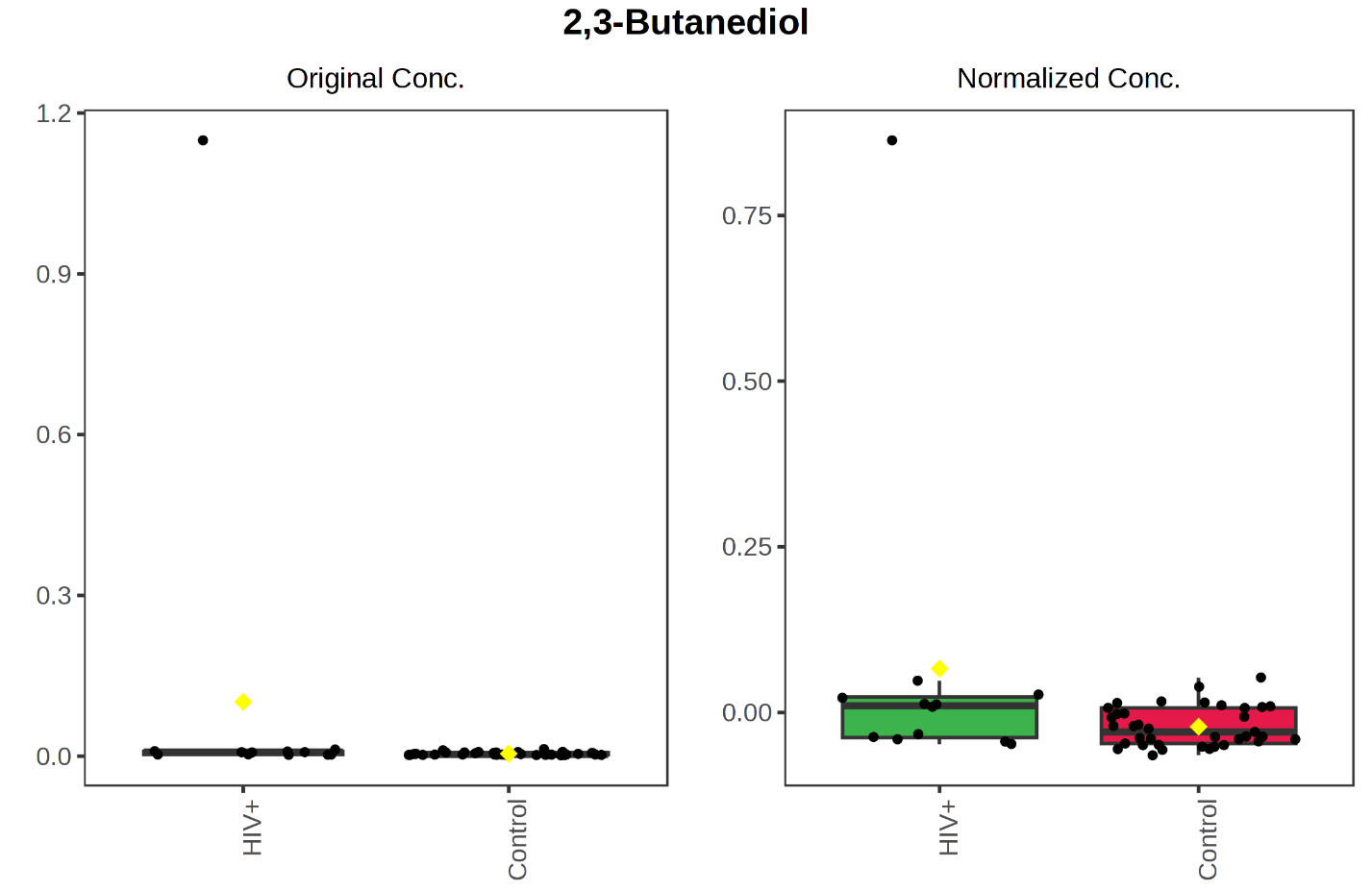


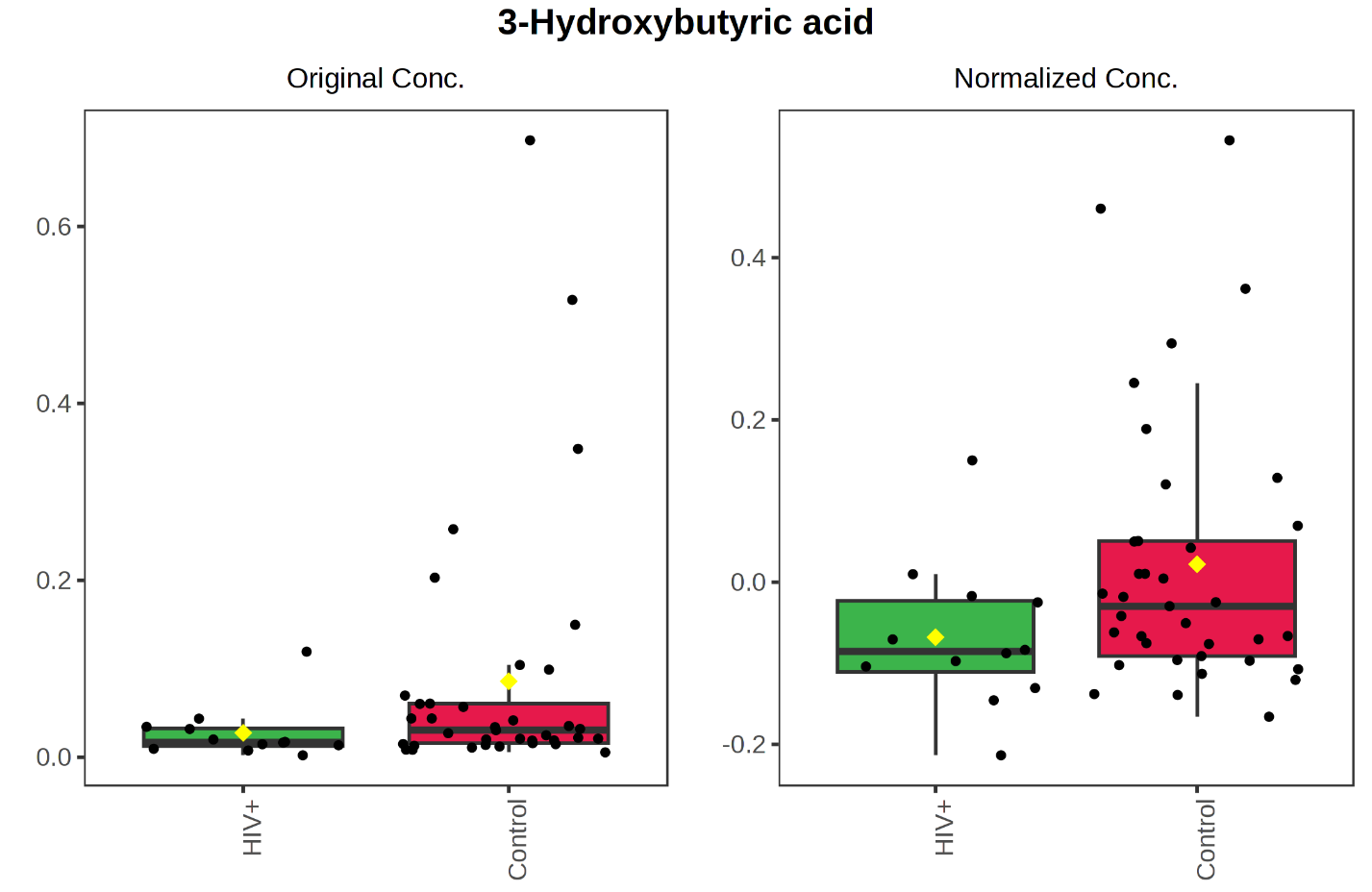

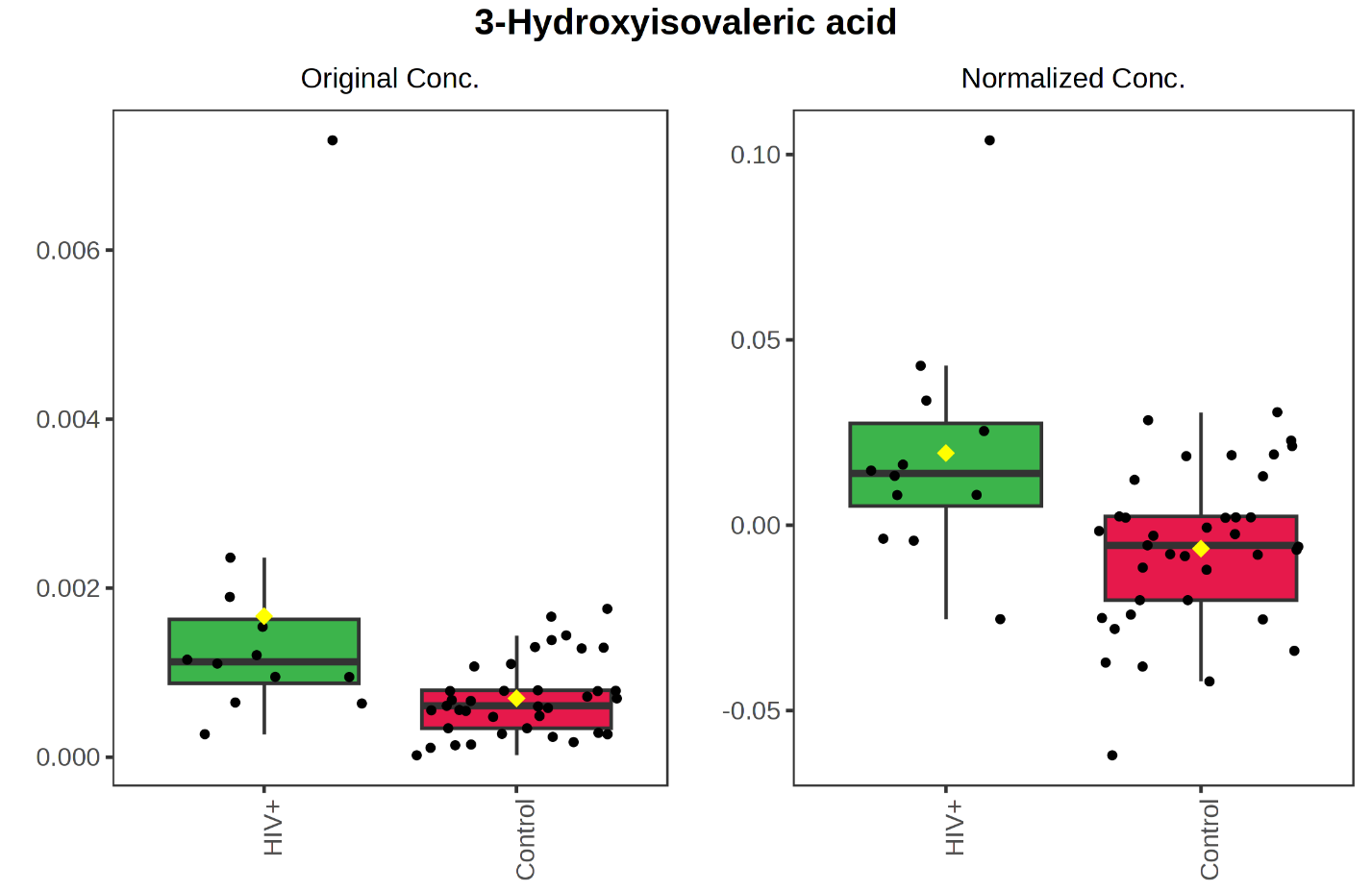

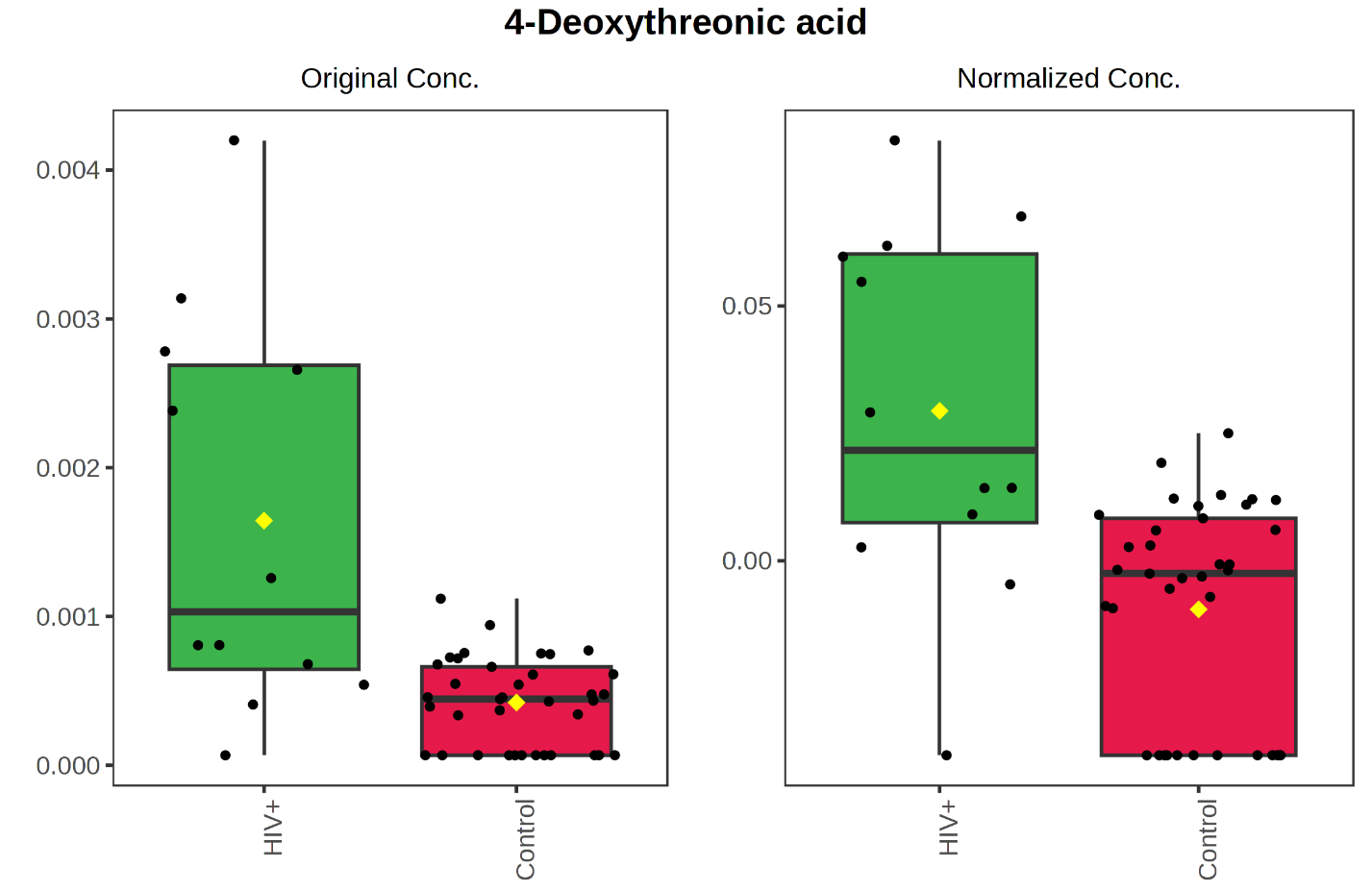

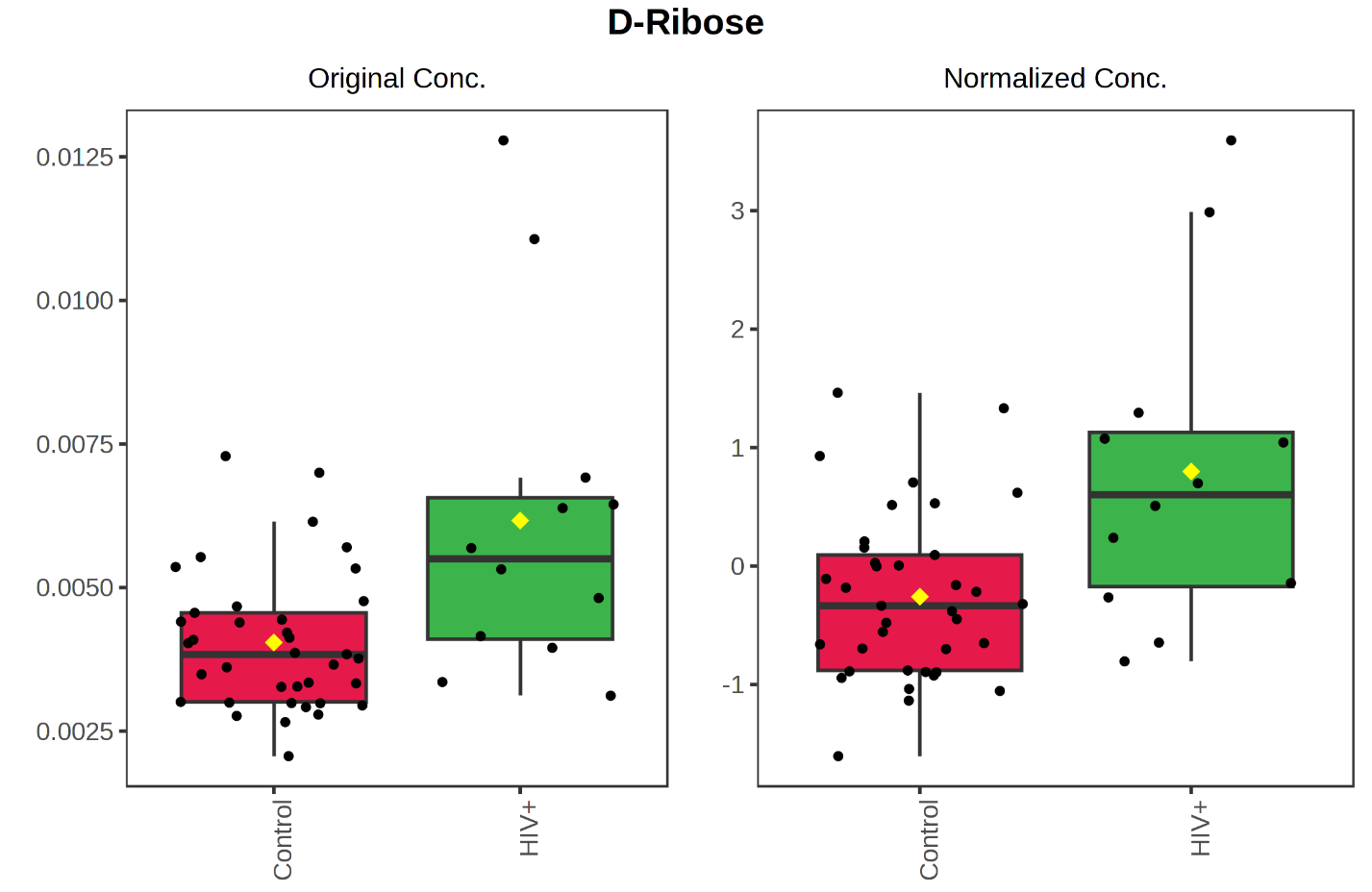

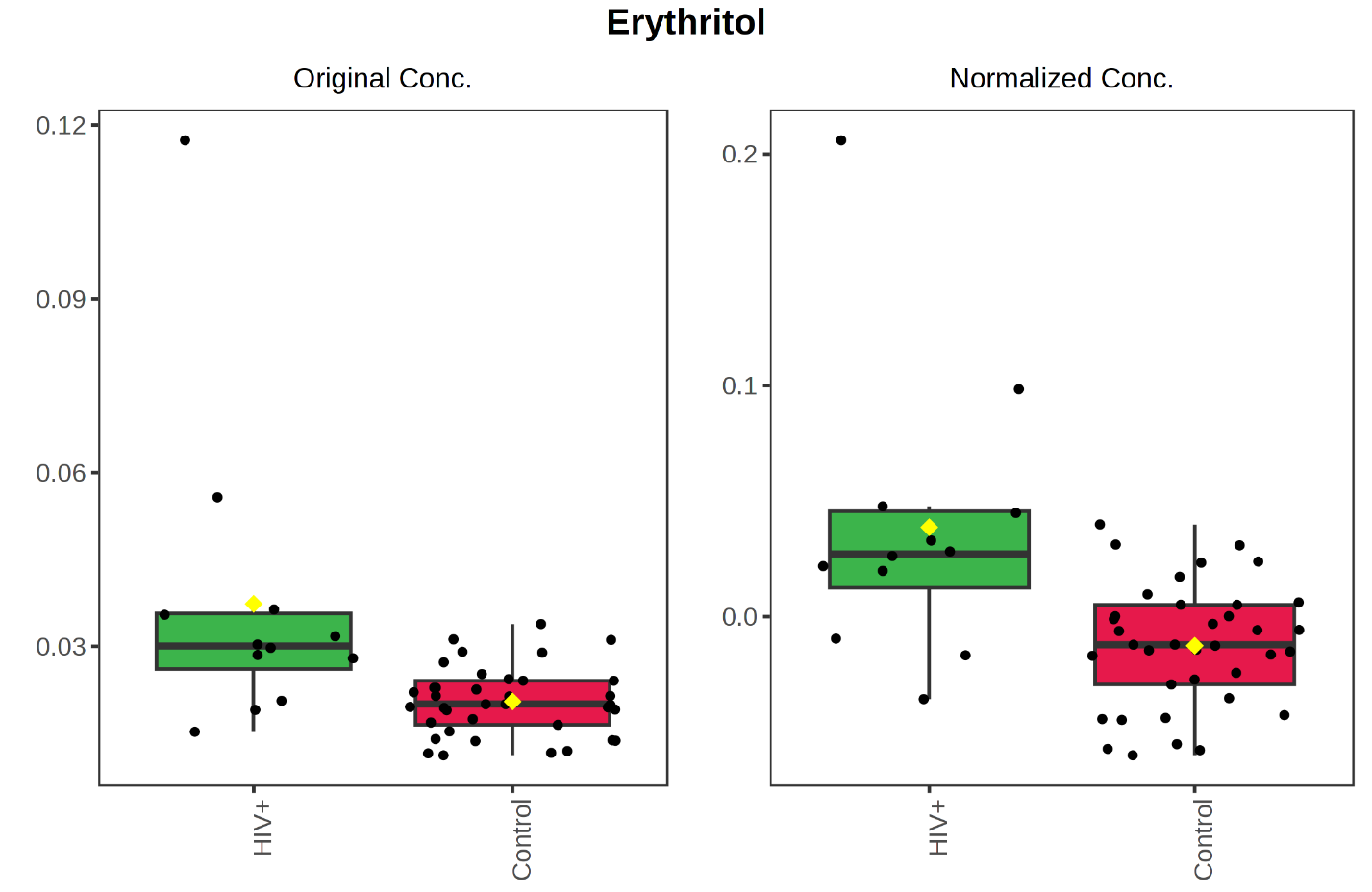

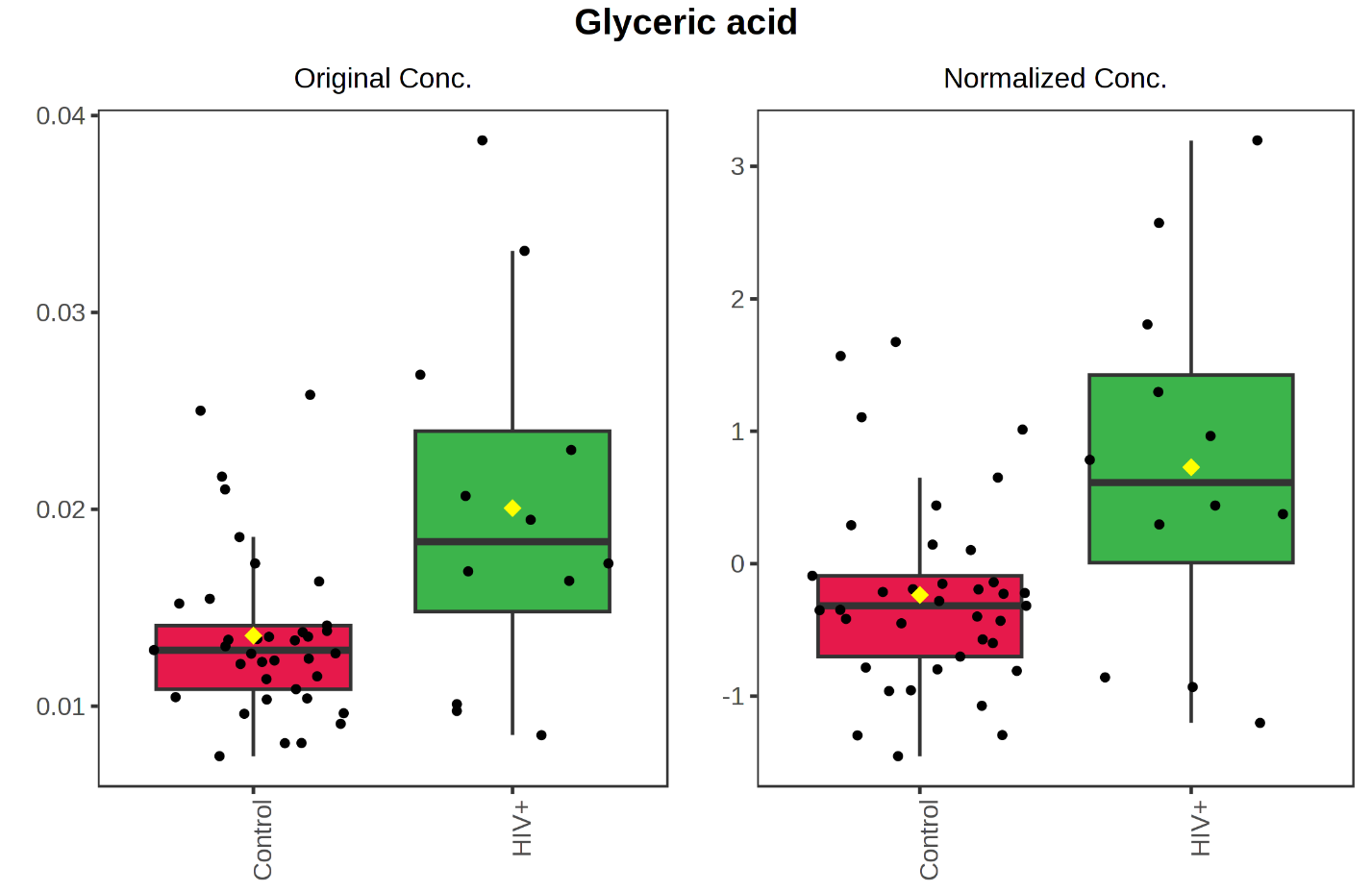

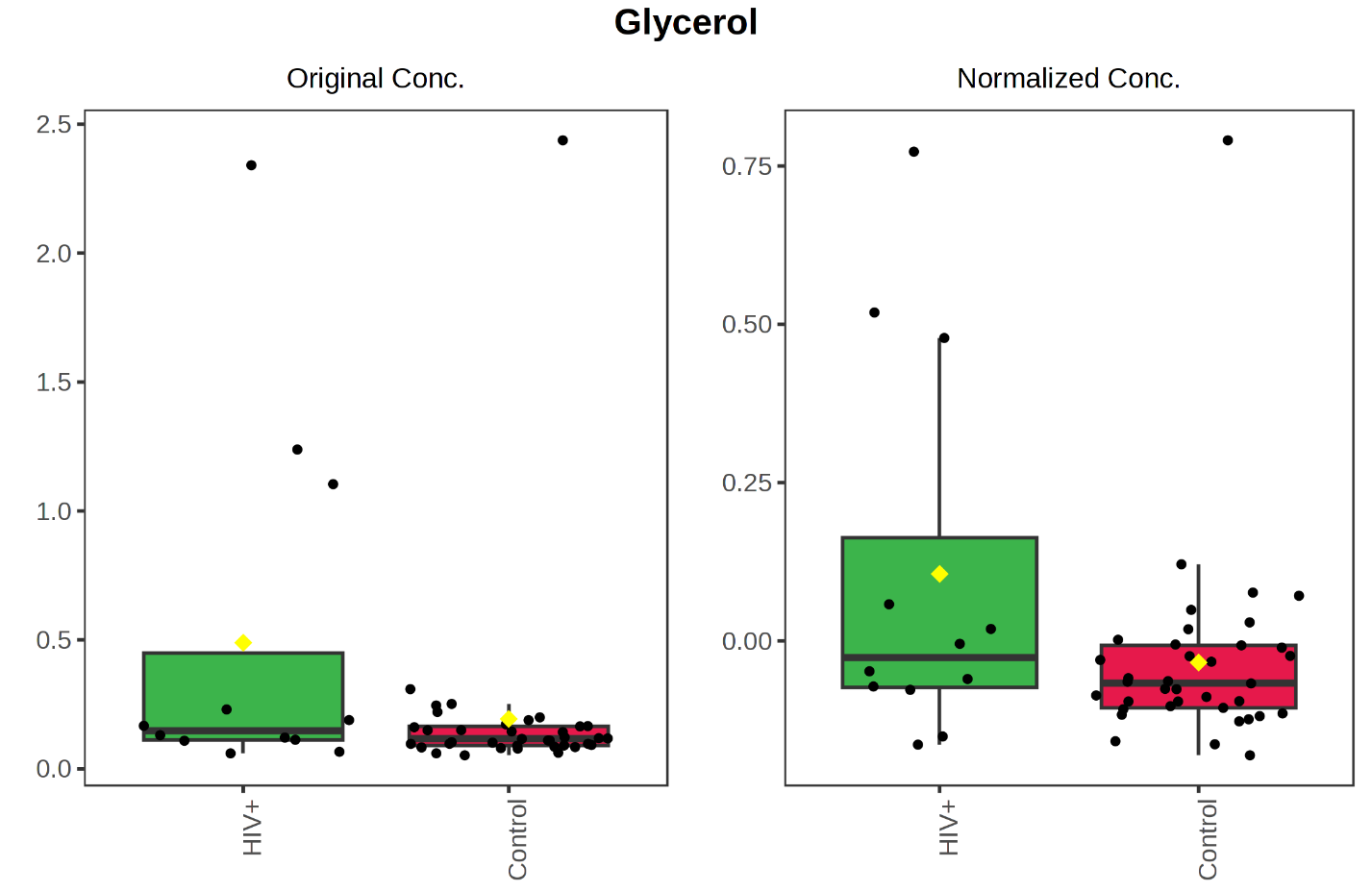

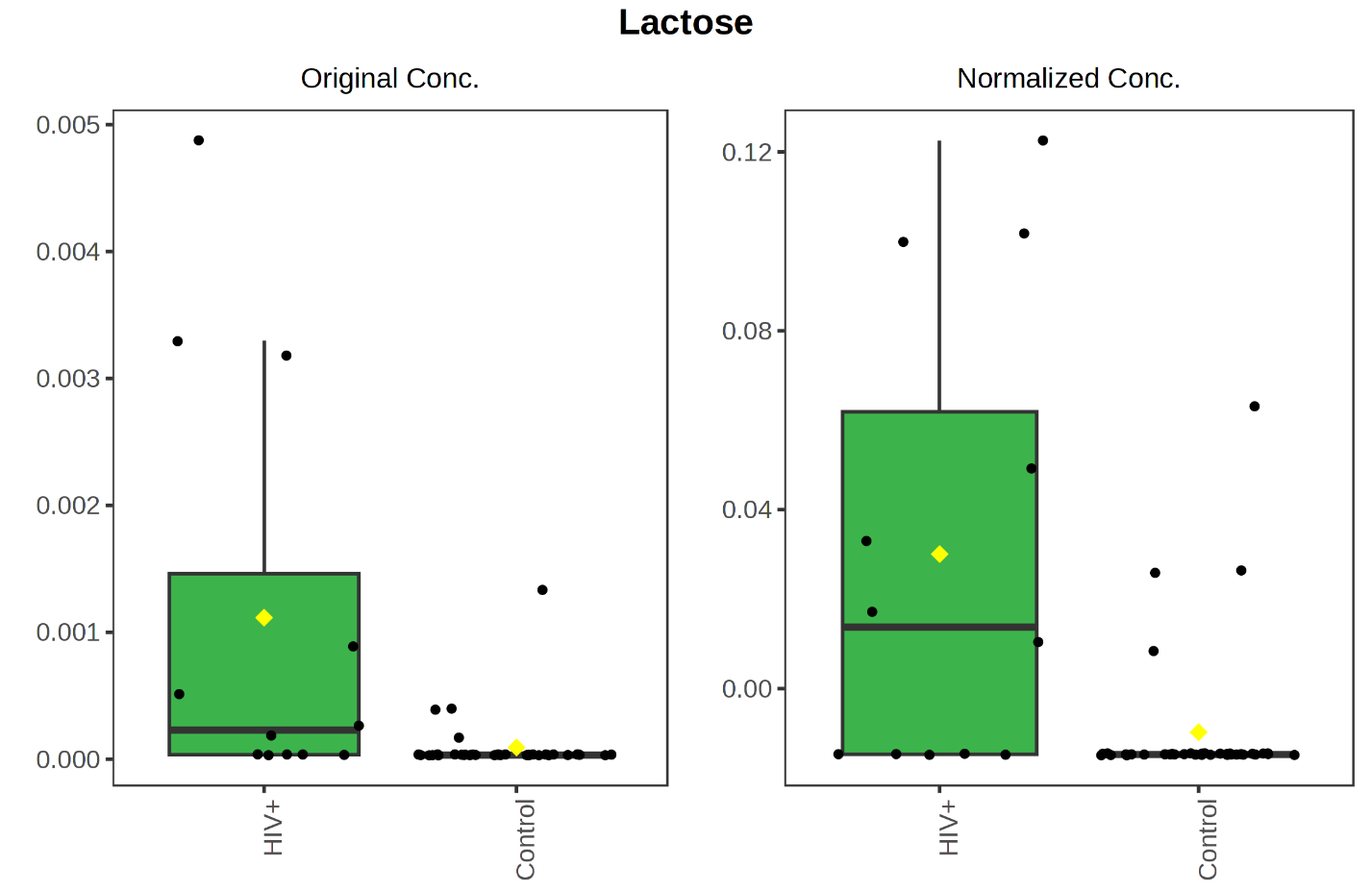

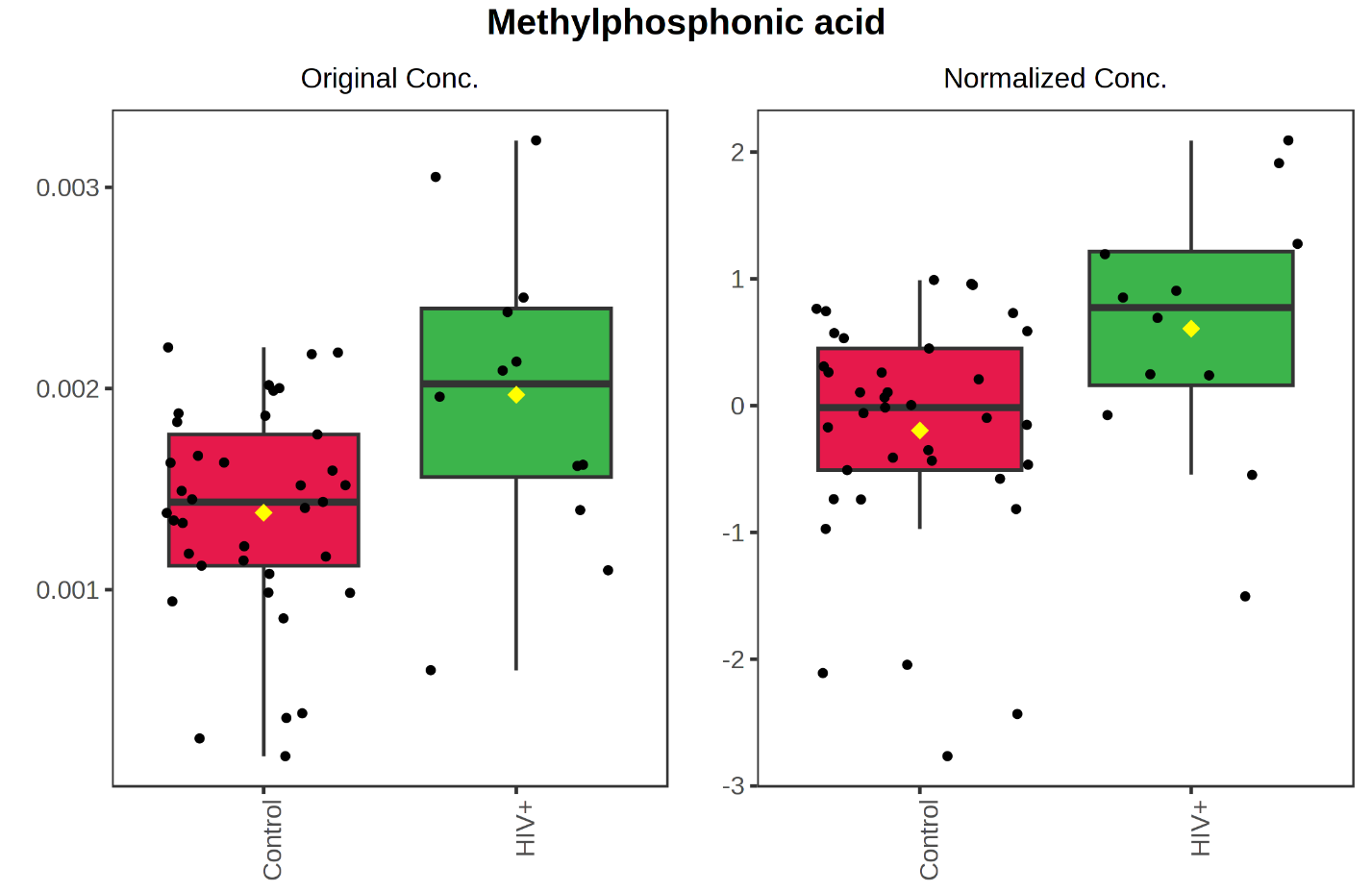

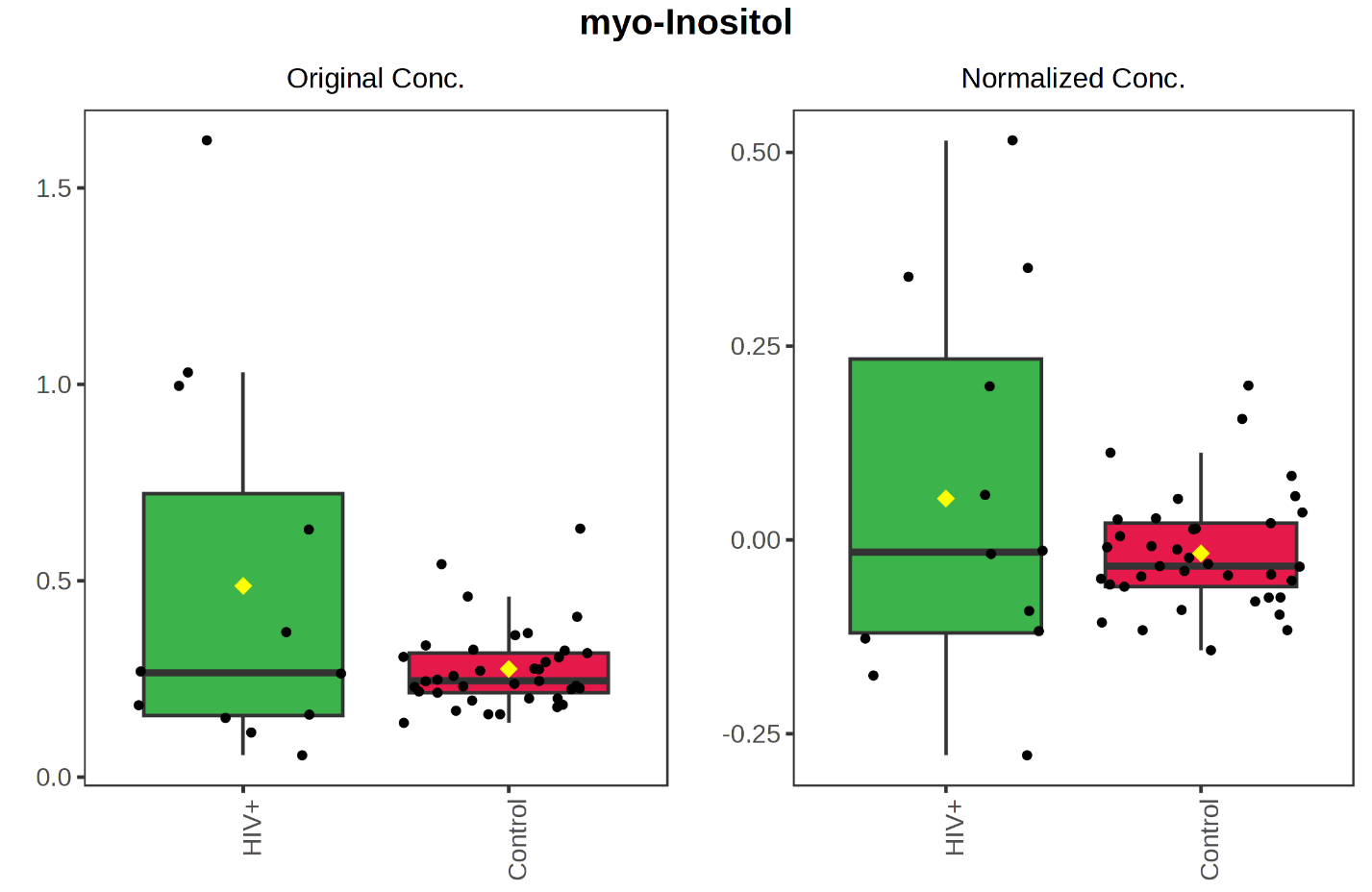

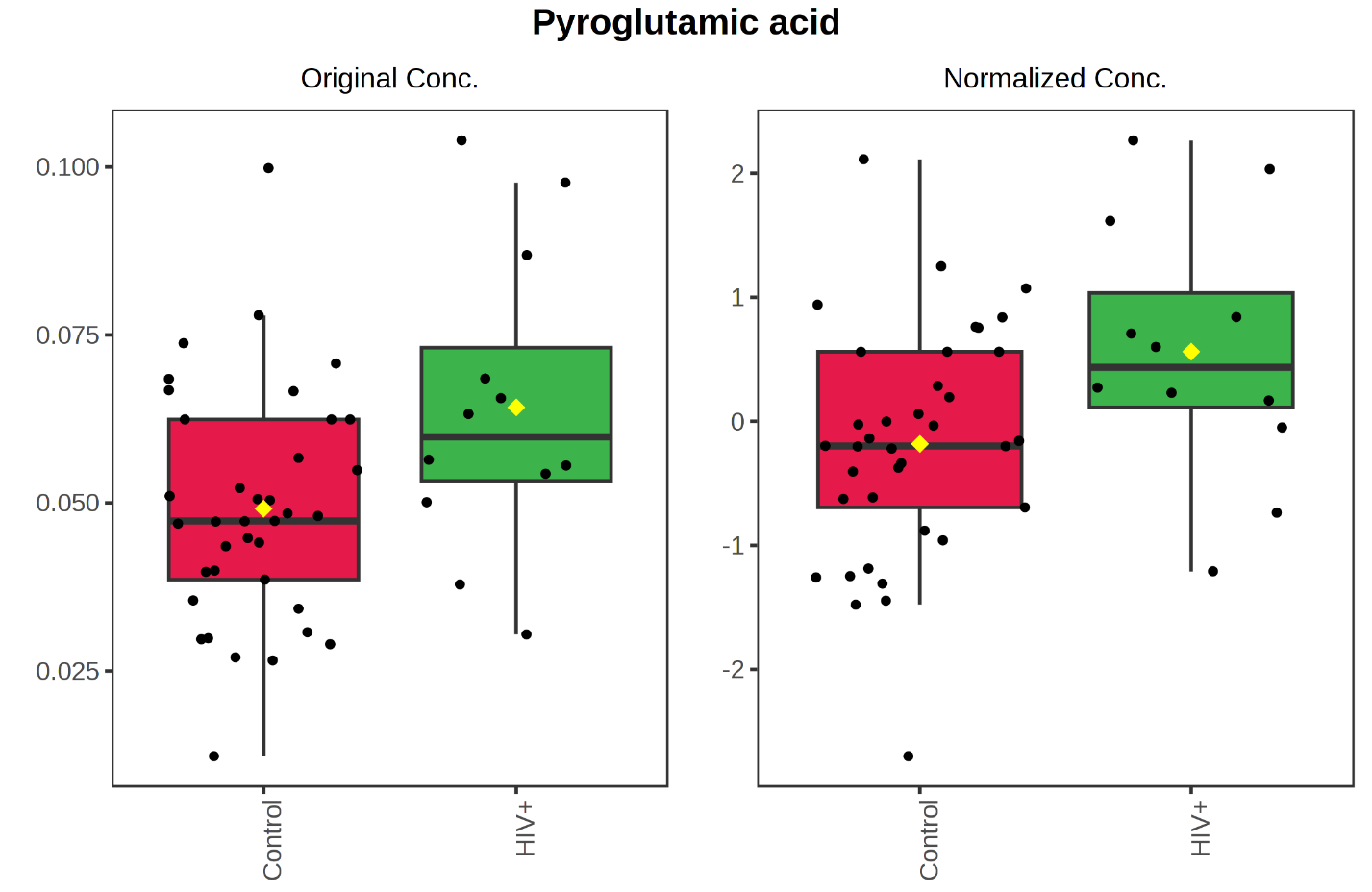

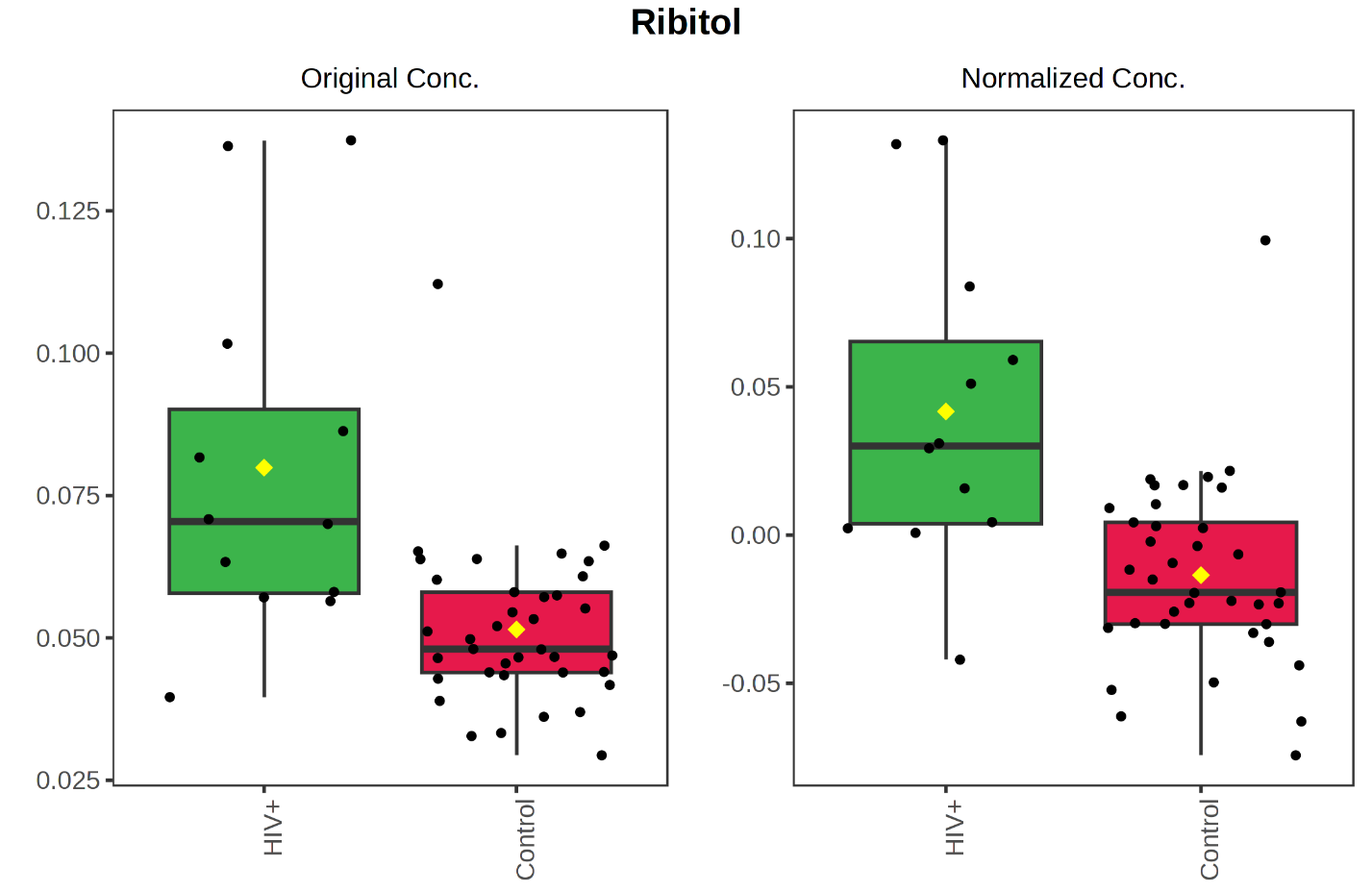

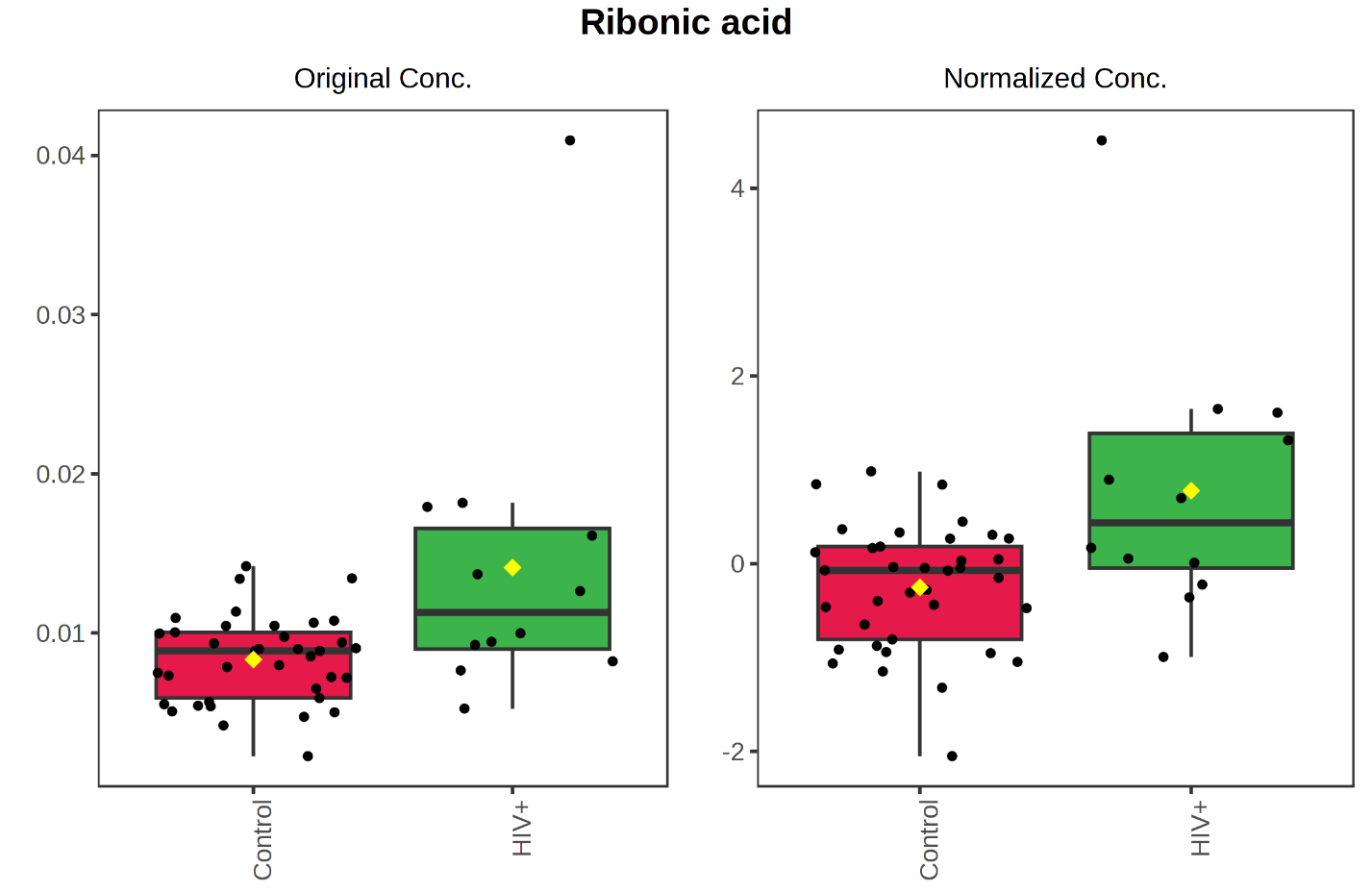

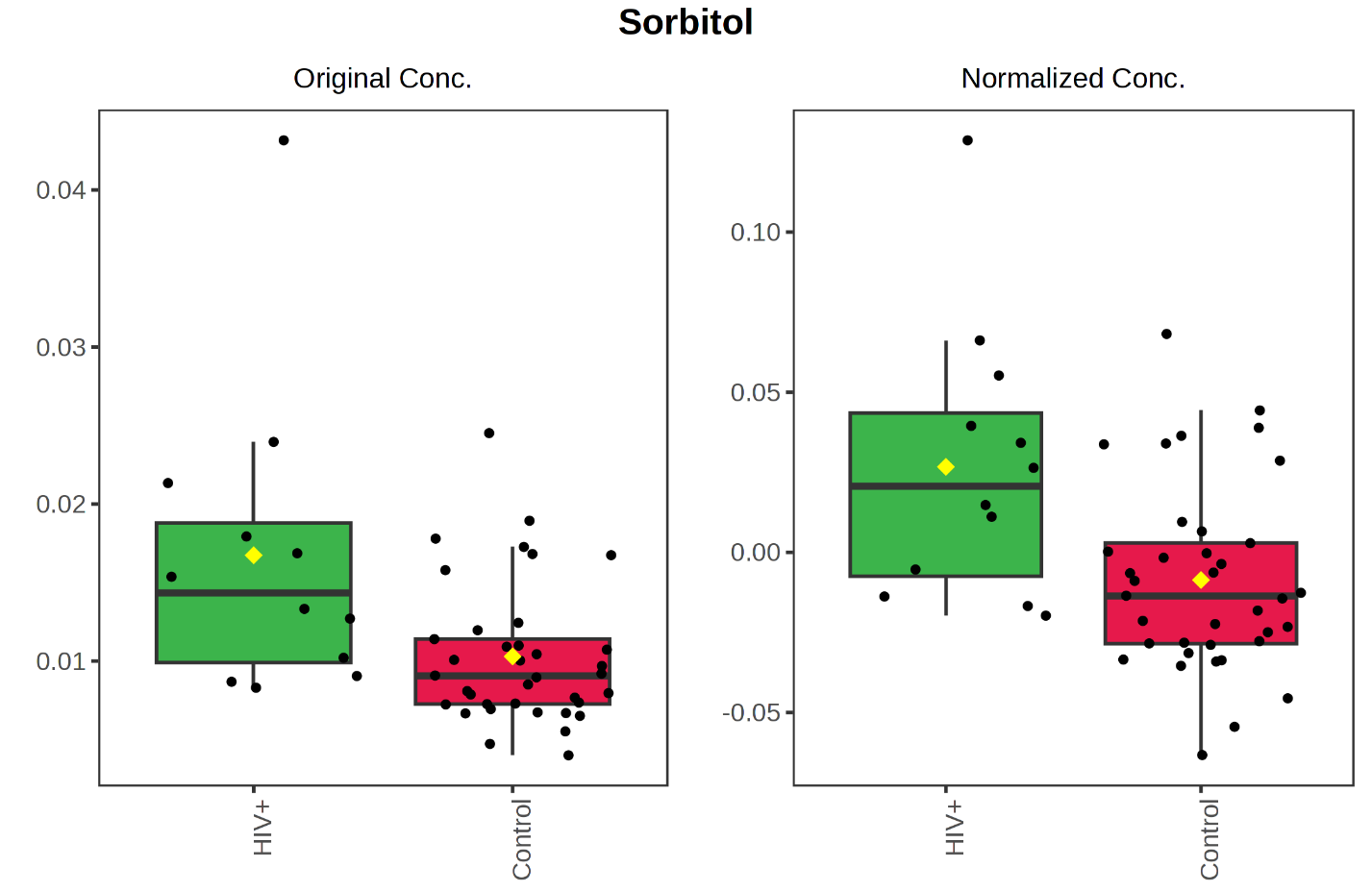

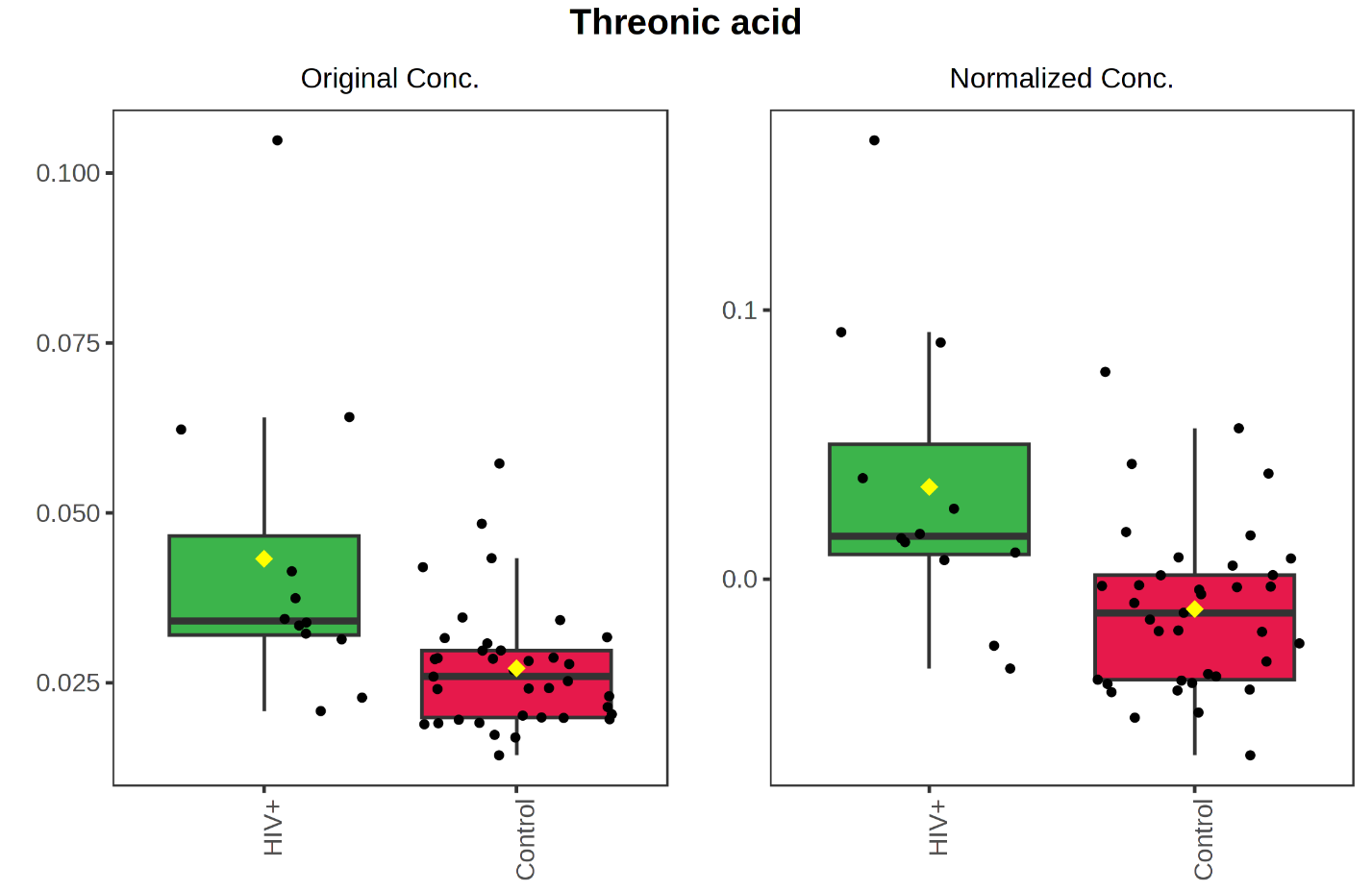

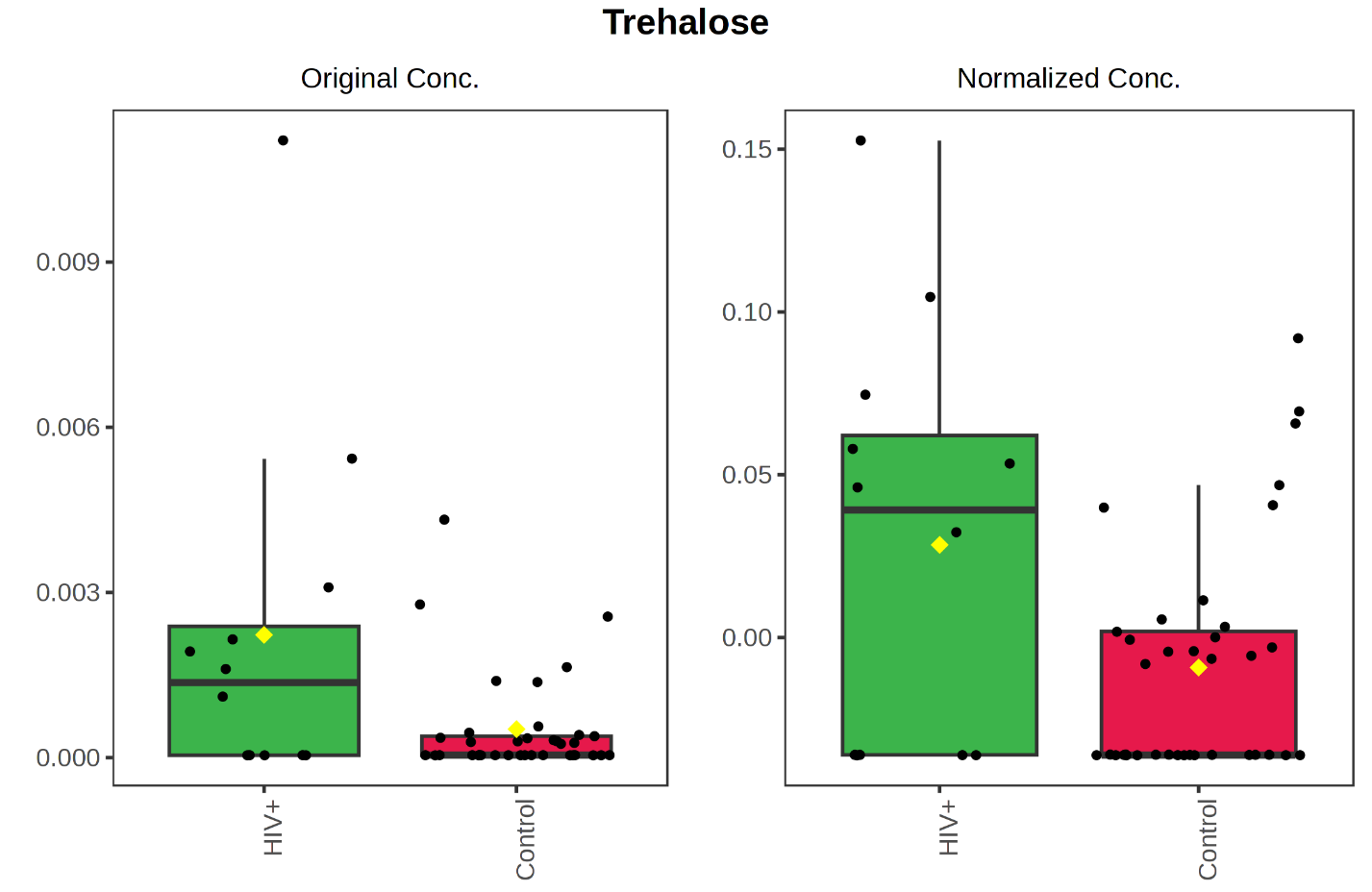

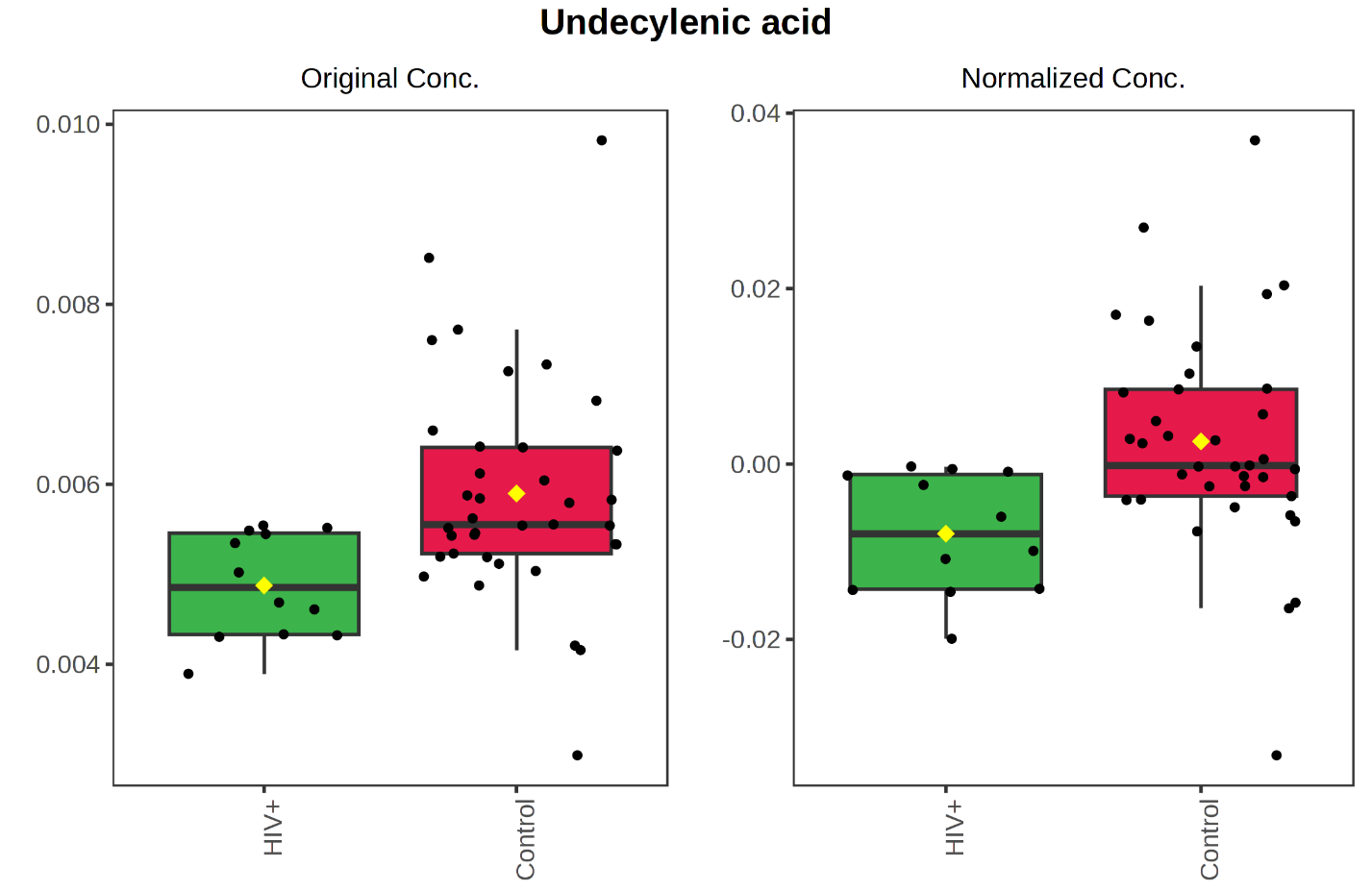

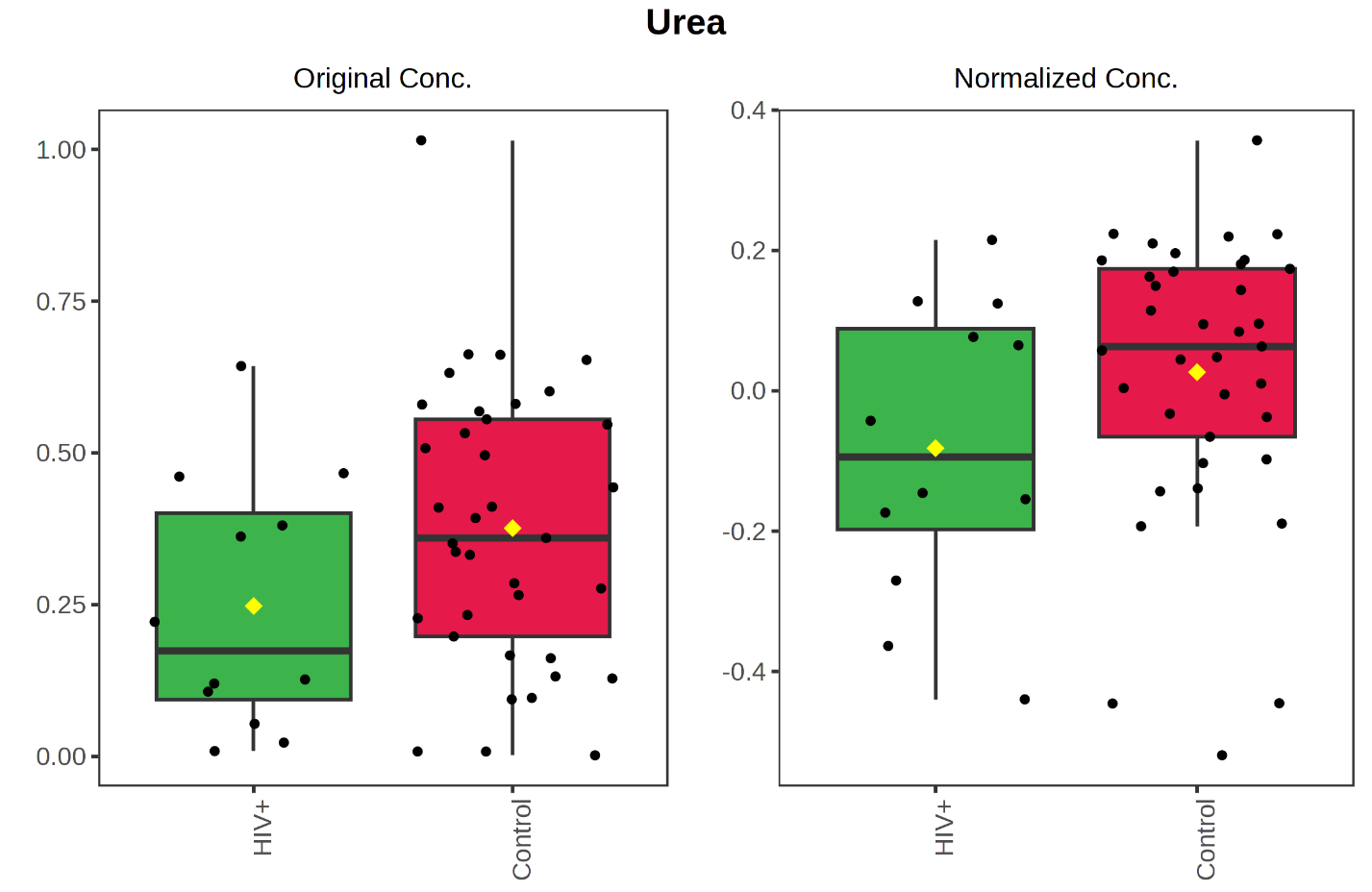

Supplement: Supplementary file 1 — Supplementary file1 (DOCX 1619 KB) [file 11306_2024_2098_MOESM1_ESM.docx]
