# Supplementary material for: An exploratory investigation of the CSF metabolic profile of HIV in a South African paediatric cohort using GCxGC-TOF/MS
Source: Metabolomics. 2024 Mar 1;20(2):33. doi: 10.1007/s11306-024-02098-y (PMC10907482; doi:10.1007/s11306-024-02098-y)
Supplement: Supplementary file 4 — Supplementary file4 (DOCX 36 KB) [file 11306_2024_2098_MOESM4_ESM.docx]

Correlation data

Table S1 and S2 reveals that there are several significant correlations among the VIP metabolites. Lactose significantly correlated with ribitol (r = 0.857; *p* = 0.014), undecylenic acid (r = 0.857; *p* = 0.014), myo-inositol (r = 0.857; *p* = 0.014), D-ribose (r = 0.821; *p* = 0.023) and pyroglutamic acid (r = 0.821; *p* = 0.023). 3-Hydroxybutyric acid significantly correlated with methylphosphonic acid (r = 0.664; *p* = 0.018). Sorbitol significantly correlated with methylphosphonic acid (r = 0.615; *p* = 0.033). Ribitol significantly correlated with threonic acid (r = 0.790; *p* = 0.002), undecylenic acid (r = 0.643; *p* = 0.024), erythritol (r = 0.832; *p* = 0.001), myo-inositol (r = 0.755; *p* = 0.005), D-ribose (r = 0.804; *p* = 0.002), ribonic acid (r = 0.769; *p* = 0.003), glyceric acid (r = 0.860; *p* < 0.0001), and pyroglutamic acid (r = 0.881; *p* < 0.0001). Erythritol also significantly correlated with threonic acid (r = 0.643; *p* = 0.024), ribonic acid (r = 0.944; *p* < 0.0001), glyceric acid (r = 0.734; *p* = 0.007), and pyroglutamic acid (r = 0.692; *p* = 0.013). Threonic acid significantly correlated with myo-inositol (r = 0.790; *p* = 0.002), D-ribose (r = 0.615; *p* = 0.033), glyceric acid (r = 0.867; *p* < 0.0001), and pyroglutamic acid (r = 0.629; *p* = 0.028). Myo-inositol significantly correlated with D-ribose (r = 0.853; *p* < 0.0001), glyceric acid (r = 0.657; *p* = 0.020), and pyroglutamic acid (r = 0.748; *p* = 0.005). Glyceric acid significantly correlated with pyroglutamic acid (r = 0.762; *p* = 0.004). Undecylenic acid significantly correlated with methylphosphonic acid (r = 0.615; *p* = 0.033). Trehalose significantly correlated with methylphosphonic acid (r = 0.821; *p* = 0.023). D-Ribose significantly correlated with glyceric acid (r = 0.622; *p* = 0.031) and pyroglutamic acid (r = 0.832; *p* = 0.001). Ribonic acid significantly correlated with pyroglutamic acid (r = 0.629; *p* = 0.028).

There were several significant correlations between the VIP metabolites and clinical features. Lactose significantly correlated with CSF glucose (r = -0.793; *p* = 0.033). 3-Hydroxybutyric acid significantly correlated CSF protein (r = 0.857; *p* = 0.014), meningeal irritation (r = 0.878; *p* = 0.009), right hemiplegia (r = 0.842; *p* = 0.009), and deceased status (r = 0.601; *p* = 0.039). Sorbitol significantly correlated fever (r = 0.668; *p* = 0.018), right hemiplegia (r = 0.796; *p* = 0.018), TB co-infection (r = 0.617; *p* = 0.033) and deceased status (r = 0.691; *p* = 0.013). D-Ribose significantly correlated with age (r = -0.632; *p* = 0.028) and infarctions (r = -0.641; *p* = 0.046). Ribitol significantly correlated with CSF protein (r = 0.821; *p* = 0.023). Ribonic acid significantly correlated with CSF protein (r = 0.929; *p* = 0.003), meningeal irritation (r = 0.800; *p* = 0.031), right hemiplegia (r = 0.804; *p* = 0.016), and deceased status (r = 0.715; *p* = 0.009). Erythritol significantly correlated with CSF protein (r = 0.893; *p* = 0.007). Myo-inositol significantly correlated with CSF glucose (r = -0.670; *p* = 0.024). Glycerol significantly correlated with left hemiplegia (r = 0.938; *p* = 0.001). 3-Hydroxyisovaleric acid significantly correlated with infarctions (r = -0.684; *p* = 0.029) and left hemiplegia (r = -0.804; *p* = 0.016). Undecylenic acid significantly correlated with CSF protein (r = 0.821; *p* = 0.023), left hemiplegia (r = -0.722; *p* = 0.043), and decreased consciousness (r = -0.615; *p* = 0.044). Methylphosphonic acid significantly correlated raised intracranial pressure (r = -0.756; *p* = 0.007), hydrocephalus (r = -0.847; *p* = 0.002), and fever (r = 0.801; *p* = 0.002). Pyroglutamic acid significantly correlated with age (r = -0.716; *p* = 0.009) and CSF protein levels (r = 0.786; *p =* 0.036).

There were several significant correlations among the clinical features. Gender correlated significantly with vomiting (r = -0.607; *p* = 0.048), in that girls were more likely to throw up. Raised intracranial pressure correlated significantly with hydrocephalus (r = 1.000; *p* < 0.0001). Hydrocephalus also correlated negatively with fever (r = -0.667; *p* = 0.035). Meningeal irritation correlates significantly with right hemiplegia, TB co-infection, and whether the patient died (r = 1.000; *p* < 0.0001). Right hemiplegia also correlated significantly with TB co-infection and deceased status (r = 1.000; *p* < 0.0001). Left hemiplegia correlated significantly with decreased consciousness (r = 1.000; *p* < 0.0001). Seizures correlated negatively with a cough (r = -0.810; *p* = 0.003). Lastly leucocytes correlated significantly with lymphocytes (r = 1.000; *p* < 0.0001).
